# Item analysis using Rasch models confirms that the Danish versions of the DISABKIDS® chronic-generic and diabetes-specific modules are valid and reliable

**DOI:** 10.1186/s12955-017-0618-8

**Published:** 2017-03-01

**Authors:** Julie Bøjstrup Nielsen, Julie Nyholm Kyvsgaard, Stine Møller Sildorf, Svend Kreiner, Jannet Svensson

**Affiliations:** 1 Copenhagen Diabetes Research Center (CPH-DIRECT), Department of Paediatrics, Herlev University Hospital, Herlev Ringvej 75, Herlev, 2730 Denmark; 2 Section of Biostatistics, Department of Public Health, Faculty of Health and Medical Sciences, University of Copenhagen, Øster Farimagsgade 5, Copenhagen K, 1014 Denmark

**Keywords:** Diabetes type 1, Children, HrQoL, DISABKIDS, Rasch, Adolescents, Chronic condition

## Abstract

**Background:**

Type 1 Diabetes (T1D) has a negative impact on psychological and overall well-being. Screening for Health-related Quality of Life (HrQoL) and addressing HrQoL issues in the clinic leads to improved well-being and metabolic outcomes. The aim of this study was to translate the generic and diabetes-specific validated multinational DISABKIDS® questionnaires into Danish, and then determine their validity and reliability.

**Methods:**

The questionnaires were translated using a validated translation procedure and completed by 99 children and adolescents from our diabetes-department; all diagnosed with T1D and were aged between 8 and 18 years old. The Rasch and the graphical log linear Rasch model (GLLRM) were used to determine validity. Monte Carlo methods and Cronbach’s α were used to confirm reliability.

**Results:**

The data did not fit a pure Rasch model but did fit a GLLRM when item six in the independence scale is excluded. The six subscales measure different aspects of HrQoL indicating that all the subscales are necessary. The questionnaire shows local dependency between items and differential item functioning (DIF). Therefore age, gender, and glycated hemoglobin (HbA1c) levels must be taken into account when comparing HrQoL between groups.

**Conclusions:**

The Danish versions of the DISABKIDS® chronic-generic and diabetes-specific modules provide valid and objective measurements with adequate reliability. These Danish versions are useful tools for evaluating HrQoL in Danish patients with T1D. However, guidelines on how to manage DIF and local independence will be required, and item six should be rephrased.

**Electronic supplementary material:**

The online version of this article (doi:10.1186/s12955-017-0618-8) contains supplementary material, which is available to authorized users.

## Background

Type 1 diabetes (T1D) has adverse effects on psychological and overall well-being [1, 2]. Research indicates that monitoring and discussing Health-related Quality of Life (HrQoL) in adolescents with T1D improves their psycho-social wellbeing [3]. However, this must be maintained as part of an ongoing process to sustain these beneficial effects in patients [4]. Quality of life is increasingly considered an important health-outcome-parameter in medicine, and the International Society for Pediatric and Adolescent Diabetes (ISPAD) recommends routine evaluations in children and adolescents with T1D [5]. To improve the quality of care and enable treatment outcomes to be compared internationally, a multinational screening method for HrQoL is needed.

DISABKIDS® started as a European-Commission-funded project aiming to develop instruments for assessing HrQoL in children and adolescents with chronic conditions. It was developed collaboratively among seven European countries: Germany (where the European DISABKIDS® coordination Group resides), Austria, the Netherlands, France, Greece, the United Kingdom, and Sweden. DISABKIDS® consists of a joint chronic-generic module (DCGM-37) and seven disease-specific modules, including a Diabetes-Specific Module (DSM-10) [6]. The other modules are specific for asthma, arthritis, cerebral palsy, cystic fibrosis, dermatitis, and epilepsy. The DCGM-37 consists of 37 questions and measures general HrQoL and the level of distress caused by a chronic disease. It explores six dimensions: independence, emotion, social exclusion, social inclusion, physical limitations, and treatment. The DSM-10 consists of 10 questions that cover two dimensions: impact and treatment.

The validity of the DISABKIDS® generic module was initially tested using factor analyses, scale score distributions, item-dimension score correlations, correlations between dimension scores, and Rasch analyses. Its reliability was tested using intraclass correlation coefficients between two successive assessments [7]. Validation of the condition-specific modules of DISABKIDS® was achieved by applying principal component analyses and tests for internal consistency [8]. The reliability of repeated measurements using the DSM-10 and DCGM-37 has been demonstrated in a Swedish population by applying intraclass correlation coefficients (ICC), Cronbach’s α, split-half reliability, and Bland-Altman plots [9]. The Norwegian translations of the DCGM-37 and DSM-10 have been subjected to explorative factor analyses [10]. However, to date, no Danish versions have been available.

The Rasch model [11–13] is an item response theory (IRT) model. A number of convenient properties within the model make it particularly appropriate for assessing summated scales. In particular, this includes the capacity for summarizing how well responses to the items can provide a measurement of a single cohesive theoretical construct, in this case quality of life.

Therefore, the aim of this study was to translate the DISABKIDS® chronic-generic module (DCGM-37) and diabetes-specific module (DSM-10) questionnaires into Danish and use the Rasch model to determine their internal validity and reliability in children and adolescents with T1D.

## Methods

Translation and validation were performed according to the “DISABKIDS® group Translation and Validation Procedure” [14] and consisted of the following steps:

- Step 1: Forward and backward translation to and from the target language was carried out according to the DISABKIDS® Group guidelines. The procedure was subsequently approved by the DISABKIDS® German study center Group.
- Step 2: Cognitive debriefing in two focus groups with either children and adolescents or parents represented. The evaluation was performed one question at a time. The purpose of this step was to ensure the consistency of the translation. All inputs were noted, considered by the research group, and alterations were made where appropriate.

### Population

Children and adolescents between 8 and 18 years of age with T1D, who were scheduled for routine follow-up at the pediatric and adolescent diabetes outpatient clinic at Herlev Hospital from July 2013 until June 2014 were included. Study participants were chosen randomly at different times during the day when they attended the outpatient clinic. Enrollment was carried out by two clinical assistants on the days they were present at the clinic. Families who were unable to speak or read Danish, patients with T1D for less than 1 month, and parents and/or children who were unwilling to participate in the study were excluded. Only the children’s responses were included in this analysis. The study sample consisted of 99 families from an entire population that included approximately 413 children and adolescents aged between 8 and 18 years old diagnosed with T1D according to the ISPAD guidelines [15].

The patient and one accompanying parent were approached during the outpatient visit. Informed consent was provided by the parents of children younger than 15 years of age and by both the parent and the adolescent for children who were at least 15 years old. The DCGM-37 and DCM-10 questionnaires were completed using an online web-based system for managing clinical trials (http://www.easytrial.net). Because the patients were encouraged to answer the questions without prompting from their parent, one of the two clinical assistants involved in enrollment was available to clarify any practical issues and help with reading difficulties in younger patients. E-mail-addresses were obtained and the re-test questionnaires were automatically distributed by email from easytrial.net 1 week later. These were completed electronically by the child at their home. A reminder was sent by email if the re-test was not completed within 4 weeks. Glycated hemoglobin (HbA1c) was measured using a high-pressure liquid chromatographic method (Tosoh Bioscience, South San Francisco, CA, USA), which had a normal operating range of 23.5–40 mmol/mol (4.3–5.8%). Patient data that included age, sex, and mode of insulin-administration (pen or pump) were recorded during the outpatient visit at the same time as enrollment.

### Ethics

The study was approved by the Danish Data Protection Agency (Region Hovedstaden 2007-58-0015). Questionnaire studies do not need ethics committee approval in Denmark.

### Statistical methods

The responses to the eight different subscales in the DCGM-37 and DSM-10 in the test and retest questionnaires were first analyzed for item fit to the Rasch model and then to the graphical log linear Rasch model (GLLRM) (16).

The items in the questionnaires have five ordinal response categories ranging from “never” to “always”. The scoring of items depends on the orientation of the questions. During the analysis, questions relating to negative experiences were coded 4 (“never”) to 0 (“always”) whereas questions relating to positive experiences were coded 0 (“never”) to 4 (“always”). A high total score therefore implies few problems and a high degree of quality of life for all subscales irrespective of the orientation of the items. The person covariates analyzed for interference in the responses were age, sex, HbA1c level, treatment module, and time.

Responses to questions collected both at inclusion and follow-up were analyzed. Repeated measurements could be included because item parameters were estimated by conditional maximum likelihood estimates in the conditional distribution of item responses, given the total scores for all items, which do not depend on person parameters under the Rasch model. For this assumption to be valid, item thresholds need to be the same at both inclusion and follow-up and this requirement was routinely tested during the analysis.

#### Assessment of significance

Significance was evaluated at a 5% critical level after adjustment for multiple testing by the Benjamini-Hochberg procedure [16]. We distinguished between weak to moderate evidence against the model when *p*-values were larger than 0.01 and stronger evidence, when p was less than 0.01 [17].

#### The Rasch model

Items fitting a Rasch model exhibit a number of properties that psychometricians sometimes refer to as criterion-related construct validity [18]. The required properties of criterion-related construct validity are: (i) unidimensionality, (ii) monotonicity, (iii) local independence, and (iv) lack of differential item functioning (DIF). For a more detailed description of these four criteria see Additional file 1. In health-related scales, it is rare to find items that satisfy the requirements of local independence and lack of DIF. In these cases, one can attempt to use graphical log linear Rasch models (GLLRM) [19–23] that relax the requirements for local independence and lack of DIF.

In addition to these four properties, items from Rasch models exhibit a fifth property called statistical sufficiency. This distinguishes Rasch model items from those of other IRT models because it means that the total score captures all the available information on the person parameter, and there is nothing more to gain by examining the pattern of responses to items once the total score has been calculated. Because of these five properties, the Rasch model justifiably embodies the type of data reduction one hopes for when attempting to construct simple and practical summated scales.

#### Assessing the adequacy of the Rasch model

To avoid assumptions on the distribution of the latent variable, the analysis in this study was based on principles of conditional inference [24–27]. This involves comparing the conditional distribution of item responses using the total score over all items with the expected conditional distributions. Because the total score is sufficient, these distributions do not depend on unknown parameters and no assumptions need to be made about the distribution of person parameters during the analysis. Our analysis assessed the overall fit of the model and the overall assessment of lack of DIF by conditional likelihood ratio tests (CLR) [26]. This tests the hypothesis that the complete set of item parameters was the same in subpopulations defined by the total score or by values of person covariates. Weak evidence against the Rasch model (0.01 ≤ p ≤ 0.05) was not regarded as conclusive if it was not supported by evidence against the fit of items, or evidence of either local dependence or DIF for specific items. To test the hypothesis that the distribution of separate items corresponded to the distribution of items in Rasch models, we used conditional infits and outfits [28, 29] and compared the observed and expected correlations between scores for separate items with the summated rest-score over all other items [28]. Finally, the assumptions of local dependence and lack of DIF were tested using conditional likelihood ratio tests [19] and by analyzing the partial association between items and exogenous variables given the total score over other items [28, 30].

#### Analysis of unidimensionality

The DCGM-37 and DSM-10 questionnaires contain items relating to eight qualitatively different functional abilities (Table 1). During the initial analysis, these were regarded as different latent dimensions. At the end of the analysis, tests of unidimensionality [21, 31] were used to confirm that the subscales relating to these functional abilities actually measure different, although correlated, latent constructs.

**Table 1:** Overview of subscales and the number of people with complete or incomplete responses to questions at inclusion and follow-up

Domain	Subscales	Questions	Orientation	Inclusion		Follow-up	
				Complete	Incomplete	Complete	Incomplete
Mental	Independence	1–6	neg–pos	88	4	51	3
	Emotion	13–19	pos–neg	86	5	50	3
Social	Inclusion	26–31	neg–pos	86	5	48	4
	Exclusion	20–25	pos–neg	87	4	53	0
Physical	Limitation	7–12	7: neg–pos, 8–12: pos–neg	86	5	52	1
	Treatment	32–37	pos–neg	82	9	47	5
Diabetes module	Impact	1–6	pos–neg	90	3	52	3
	Treatment	7–10	pos–neg	92	1	52	3

The number of completed questionnaires from the 99 participants

#### Analysis of reliability

In classical test theory, reliability is either defined as the ratio between the variances of true and observed scores or by the test-retest correlation under the assumption that repeated responses to items depend only on the underlying latent construct, but not directly on previous responses to the same items. Two problems follow from these definitions:

The first problem is that reliability depends as much on the study population as on the measurement instrument, because the variances and the correlations refer to variances and correlations in data. If the variance differs between different populations, it therefore follows that the reliabilities also differ between these populations. DIF among items will also influence the distribution of the true scores. Therefore, reliability has to be calculated separately for all groups defined by values of variables with DIF effects.

The second problem is that reliabilities cannot be estimated directly in data, because the true scores are unobservable. Two assumptions regarding the test-retest experiments must be true: 1) the latent variables are unchanged; 2) at retest the respondents have no recollection of their responses at the initial test. However, this expectation is unrealistic.

To overcome the second problem, Cronbach’s α is often used as a conservative measure of reliability because it is known that Cronbach’s α provides a lower limit to the true value of reliability if it is fair to assume that items are locally independent. In case of violation of this assumption the Monte Carlo method proposed by Hamon & Mesbah [32] can be used to calculate unbiased estimates of the true reliabilities. The Monte Carlo method assumes that the distribution of person parameters is normal. Hamon & Mesbah’s procedure estimates the mean and the variance of this distribution and generates a random sample of person parameters from the distribution with two sets of random responses to the items by assuming that responses come from the Rasch model and only depend on the generated person parameter. With this data it is possible to estimate the variance of both the observed and expected total scores over all items, and also the correlation of repeated measurement assuming that the respondent has no recollection of their first response to the items when they respond the second time. In our study, we generated 10,000 random participants with repeated responses to the items and reported the estimates of reliability in separate age-and-sex groups to account for DIF and differences in score distributions among the different groups. Cronbach’s α was also reported for comparison. However, because the assumption of local independence may not be applicable α may provide an overestimate of reliability.

#### Targeting

A fundamental property of Rasch models is that person parameters and item thresholds have values on the same parameter scale. A study population is outside the target range if the range of person parameters are not included in the range of item parameters, because person parameter estimates may be biased and have very large measurement standard errors in this case. For good targeting, it is not essential that the distributions of person and item parameters are identical. However, the majority of participants should be included in the range of item parameters and the distribution of item thresholds should not be too skewed toward either low or high person parameter values. During a Rasch analysis, targeting is often assessed using item maps that compare these distributions and the item maps are provided with calculations of the average bias and average standard errors of measurement as Additional file 1 in this study.

#### Statistical software

The item analysis by Rasch models and GLLRMs was performed using DIGRAM [29, 33].

## Results

A total of 99 children took part in the study and three additional children were included but did not respond to any questions. Fifty-eight children responded to DCGM-37 and DSM-10 questions at both inclusion and follow-up. One child responded to the questions at follow-up but not at inclusion. Therefore, the item analysis included a total set of 158 responses to items. As described above, because conditional inference was used, person parameters could be excluded from the analysis of fit for item responses. During this part of the analysis, we may therefore treat two sets of responses from the same child as if they were two sets of responses from different children. The lists of DCGM-37 and DSM-10 questions are provided as Additional file 1. Table 1 provides information on the subscales together with information on the number of children who provided complete and incomplete responses to the questions. The ages of the children ranged from 8 to 18 years old and the mean age was 13.1 years. A total of 49% of the children were male. The mean duration of T1D was 31 months and the mean HbA1c level was 60.3 mmol/mol. In total, 46.4% of the children used insulin-pen injections, while the others used insulin-pumps.

### Validity

The Independence scale was chosen as an example for the results section in the following description. Additional results from the analysis of the Independence scale and the results for the rest of the DISABKIDS® subscales can be found in the Additional file 1.

The overall test-of-fit (using CLR tests) of the Independence scale (items 1–6 in DCGM-37) of the Rasch model rejected item homogeneity and suggested some degree of DIF was present (Table 2A). The model further suggested that item number six (“Are you able to do things without your parents?”) was problematic because there were highly significant differences between the observed and expected item-rest-score correlations (Table 3A). Because the observed correlation between item six and the remaining independence items was much weaker than expected, we assumed that item six probably does not measure independence, but instead a factor that is statistically related to independence. The Rasch model was therefore rejected and the final analyses of the Independence scale were carried out without item six [23]. Subtracting item six improved the fit of the Rasch model, but the model was still rejected by the CLR test of item homogeneity (*p* = 0.003). The test-retest of the scale demonstrated no signs of DIF relative to inclusion or follow-up (CLR = 22.9, df = 22, *p* = 0.405; Table 2A).

**Table 2:** Test-of-fit for two models using conditional likelihood ratio tests and comparing estimates of item thresholds in groups defined by different test criteria

Test criterion	A: Rasch model–six items			B: Graphical log linear Rasch model^a^–five items		
	CLR	Df	*P*	CLR	df	*p*
Low and high score groups	47.6	22	.001	34.3	33	.407
HbA1c	54.5	44	.133	90.0	66	.026
Treatment	42.3	22	.006	49.8	33	.030
Age	68.7	44	.010	65.3	44	.020
Sex	32.3	22	.072	36.6	33	.306
Inclusion or follow-up	22.9	22	.405	35.5	33	.350

The pure Rasch model is rejected because of local dependency between items 1 and 3 and because it operates differently for different age groups and treatments; however, it is improved when item 6 is excluded and GLLRM is applied allowing for local dependency between items 1 and 3 and DIF in relation to age

^a^The model assumes that items 1 and 3 are locally dependent and that item five is affected by DIF depending on age

**Table 3:** Item fit statistics comparing the observed and expected item-rest-score correlations under the two models

Item	A: Rasch model–six items			B: Graphical log linear Rasch model^a^–five items		
	Observed γ	Expected γ	*p*	Observed γ	Expected γ	*p*
CG1	.54	.58	.48	.56	.63	.24
CG2	.62	.57	.39	.65	.69	.41
CG3	.73	.58	.017	.74	.62	.051
CG4	.68	.59	.12	.70	.70	.99
CG5	.71	.58	.052	.74	.67	.17
CG6	.27	.56	< .001			

The correlations are measured using Goodman and Kruskal's gamma. Goodman and Kruskal's gamma measures rank correlation for ordinal categorical data [37]. When item 6 is excluded, and local dependency and DIF are allowed the observed correlations are the same as expected

^a^The model assumes that items 1 and 3 are locally dependent and that item five is affected by DIF depending on age

The CLR test for local dependence testing all pairs of items, and for DIF testing all combinations of items and covariates [19] provided significant evidence of local dependence between item one (“Are you confident about your future?”) and item three (“Are you able to do everything you want to do even though you are ill?”) (CLR = 35.7, df = 16, *p* = 0.003). Furthermore, item five (“Are you free to lead the life you want even though you are ill?”) showed evidence of DIF relative to age (CLR = 23.4, df = 8, *p* = 0.003). Local dependency is presented for all subscales in Additional file 1.

The results presented in Table 3 suggest there is a monotonic relationship between responses to items and the underlying latent variables, because the correlations between separate items and total scores over all other items (assumed to depend on the latent trait) are positive.

**Fig. 1:**
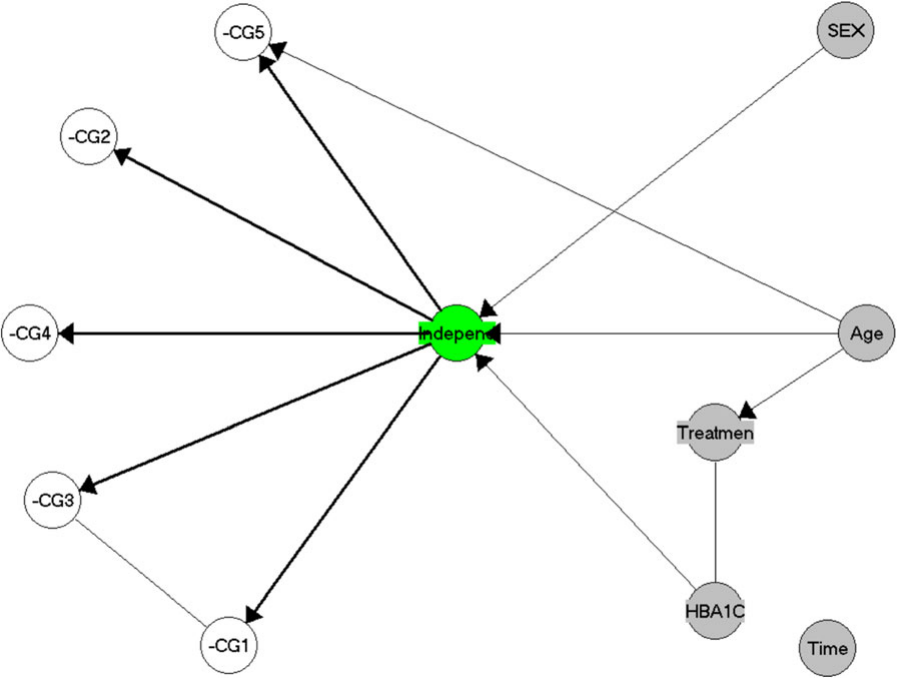
Graphical representation of the item response theory (IRT) for the GLLRM model. Item response theory (IRT) graph showing the relationships between items (CG1-5), independence (*green*), and background variables (*grey*). The arrows and edges between the covariates indicate that these variables are statistically associated. The IRT graph includes information on local dependence (e.g., line between CG1 and CG3), differential item functioning (DIF) (e.g., arrow between age and CG5), and the effect of background variables on independence (e.g., arrow between HbA1c), age, sex, and independence). Whereas time and treatment do not display any DIF or effect on independence

Further analyses were carried out applying the overall tests-of-fit and item statistics under a GLLRM (Fig. 1, Tables 2B and 3B). This is illustrated in Fig. 1 represented as a chain graph model. Here, a missing connection between two variables indicates that the variables are conditionally independent, given other variables in the model. An undirected edge between two variables indicates that the variables are conditionally dependent without assuming a causal relationship. Finally, an arrow indicates a causal relationship. Therefore, Fig. 1 indicates that the latent independence variable contribute to responses to all items, that items CG2 and CG4 are locally independent while items CG1 and CG3 are locally dependent, and that age has a DIF effect on item CG5 in addition to an indirect effect mediated by independence.

The over-all test-of-fit to the GLLMR indicates marginally significant results for lack of DIF with respect to HbA1c level, treatment, and age, but adjustment for multiple testing dismisses these results as unconvincing (Table 2B). Items one and three were locally dependent (Table 4) and age was a source of DIF for item five.

**Table 4:** Overview of results for all subscales

Domain	Subscales	Items	Local dependence	DIF	CLR	df	*p*
Mental	Independence	1–5	Items 1 and 3	Item 5–Age	34.3	33	.41
	Emotion	13–19	None	Item 13–Sex, Item 19–Age	35.3	33	.36
Social	Inclusion	26–31	Items 28 and 29	None	25.0	28	.63
	Exclusion	20–25	None	Item 21–Age, Item 22–Age & HBA1C, Item 24–Sex	44.4	42	.37
Physical	Limitation	7–12	None	Item 8–Age	29.2	28	.40
	Treatment	32–37	Item 33 and 35	None	28.8	32	.63
Diabetes module	Impact	1–6	Items 1 and 2	None	39.8	32	.16
	Treatment	7–10	None	Items 7, 9, 10–Age	26.2	38	.93

This overview includes information on the local dependence and DIF for the graphical log linear Rasch models that fit the data. The CLR test is the conditional likelihood ratio test comparing item parameters among children with high or low scores on the subscales

Table 4 shows the overall tests of the Rasch model for the other seven subscales. Because there was a fit to GLLRMs, taking local dependence and DIF into account, we conclude that the analysis supports the claim that measurement using the DISABKIDS® subscales is essentially valid and objective.

Finally, Table 5 shows the results of the analyses for unidimensionality of subscales belonging to the same domain. During these analyses, we calculated the expected correlation of the subscales on the assumption that they all measure the same latent variable, and compared the expected and observed correlations using the data. If the observed correlation was weaker than the expected correlation we concluded that one latent variable was insufficient to explain the correlation between the subscales (i.e., more than one latent variable is required). Since the tests reject unidimensionality for every pair of subscales (Table 5), the analysis suggests that the DISABKIDS® domain is composed of qualitatively different but correlated latent constructs.

**Table 5:** Overview of the results of testing for unidimensionality with subscales belonging to the same domain

Domain	Subscale 1	Subscale 2	Observed correlation	Expected correlation	*p*
Mental	Independence	Emotion	.62	.71	.009
Social	Inclusion	Exclusion	.43	.79	< .001
Physical	Limitation	Treatment	.51	.58	< .001

None of the tests support unidimensionality indicating that the subscales represent two different aspects of each domain

### Reliability

Cronbach’s α was 0.81 for the original six independence items and 0.83 for the five items included in the final model (Table 6). Regulation for dependence and DIF was included so that the estimates were calculated in separate age and sex groups. The true reliability for the youngest girls was 0.72 with relatively little variation. For all other groups, reliabilities were distributed within the 0.81–0.91 interval. The test-retest reliability was also calculated by correlation to the observed test-retest (final column Table 6).

**Table 6:** Overview of the reliability of subscales

Domain	Subscales	Cronbach’s Alpha	Reliability^a^	Reliability depends on	SEM^b^	Observed test-retest correlation
Mental	Independence	0.83	0.72–0.91	Sex and Age	1.5–1.8	0.73
	Emotion	0.82	0.65–0.89	Sex and Age	2.6–3.0	0.64
Social	Inclusion	0.64	0.64–0.66	Sex and Age	2.6	0.57
	Exclusion	0.75	0.50–0.84	Sex, Age, and HbA1c	2.2–2.7	0.85
Physical	Limitation	0.71	0.67–0.78	Age	2.4–2.5	0.57
	Treatment	0.80	0.79	None	3.0	0.69
Diabetes module	Impact	0.78	0.77–0.78	Sex	2.4	0.69
	Treatment	0.84	0.82–0.88	Age	1.5–2.0	0.77

This table displays both Cronbach’s Alpha, which is known to provide a lower bound to the true reliability if items are locally independent, and reliability calculated using the Monte Carlo method. The observed test-retest results are provided in the final column

^a^Reliability [Variance (True score)/Variance (Score)] depends on both the population and on the DIF among items. It is necessary to calculate reliability in subgroups defined by variables with a significant effect on the score. Reliability is therefore reported as an interval from the smallest to the largest degree of reliability in these groups

^a^SEM = The standard error of the total score as an estimate of the true score. The SEM depends on the true score and the DIF. SEM is therefore reported as an interval of the largest SEM value in the groups defined by the sources of DIF

The target score differed according to age, with a higher degree of targeting among the older children, where the average amount of test information provided by the independence items was 79% of the maximum obtainable information. The lowest degree of targeting was observed in the youngest children where independence items only provided 59% of the possible information required for perfect targeting. However, the overall study population was not significantly off target and was therefore adequate. Further details are included in the Additional file 1.

## Discussion

Overall the Danish translations of DISABKIDS® DCGM-37 and DSM-10 demonstrated good validity and reliability. This was the case when item six from the Independence scale was excluded and the GLLRM allowed for local dependency and DIF.

### Item six

During the development and validation of the DISABKIDS® DCGM-37, the Rasch test had been applied and lack of correlation of item six to the rest of the Independence scale was not reported. Other DISABKIDS®-translation studies have not raised this issue either [9, 10]. Although the Danish translation is grammatically correct, meanings may differ between cultures and the item’s meaning may not be appropriate in a Danish setting. The explorative factor analyses applied to the Norwegian translation [10] is not suitable for detecting lack of correlation within a scale. Because Denmark and Norway are considered very similar countries, applying Rasch analyses to the Norwegian data could prove an interesting way to test the function of item six in the Norwegian population. If the Danish translation is the problem, the item could be rephrased or replaced by the question “Are you able to do the same things, as your friends at your age, without your parents?”, although this would require a new validation procedure. Because the item fit statistics accept the fit of items to the model, we conclude that the model provides an adequate fit to the first five independence questions. However, our current statistical analyses suggest that item six should be excluded from the Danish questionnaire.

### Local independence

The Rasch test identifies a number of items with local dependency, meaning that the answer to one question is dependent on the answer to another question in the same scale. If local dependency was widespread, this would reduce the power of the items. However, because the total scores were statistically sufficient for both the pure Rasch model and the GLLRM, this local item dependency was not a major problem and the final scores still reflected the underlying HrQoL. Local dependency will influence reliability and therefore Cronbach’s α provides an overestimate of the true reliability.

### Differential item functioning

DIF is present when the response to a given item varies because the respondents are from different groups (e.g., different age, sex, and/or HbA1c level). DIF was evident for all subscales except “Social inclusion scale”, “Physical treatment scale”, and “Diabetes impact” (Table 4). This is not surprising because previous research has shown that HrQoL is influenced by age, sex, and HbA1c level [34, 35]. To compare total scores among patients from different age groups, sex, or Hba1c level, the DIF has to be taken into consideration and appropriate adjustments made. The DIF analysis also demonstrated that items produced similar responses at inclusion and follow-up, which is consistent with results from a research group in Sweden [9]. Therefore, implementing annual screening of HrQoL using the DISABKIDS® DCGM-37 and DSM-10 in a clinical setting is feasible because changes in DISABKIDS® scores will reflect changes in HrQoL without risk of confounding due to ‘item drift’. A statistical analysis of the relevant variables can be found in the Additional file 1.

### Unidimensionality

Unidimensionality was confirmed within the subscales, but rejected between the subscales. This result is consistent with previous analyses performed during the development of the questionnaire [7]. Because unidimensionality was confirmed internally, criterion-related construct validity can be confirmed. However, because there was no unidimensionality between the subscales, reduction to a single subscale would be invalid. From a clinical point of view, this means that each subscale has to be considered individually when assessing a patient’s quality of life. The lack of unidimensionality between subscales strengthens the validity of the questionnaire and confirms that different aspects of HrQoL are actually being measured.

### Reliability

As in similar studies performed previously, the reliability was satisfactory. The fact that Cronbach’s α increased when item six was withdrawn from the Independence scale indicated that this item did not influence the score. A Norwegian study group demonstrated that Cronbach’s α was low for the subscales “Physical limitation” and “Social inclusion” [10], although this was not reproduced in our data or during the development of the questionnaires. This discrepancy may be due to cultural differences, differences in the translations, or in the statistical methods applied. The transcultural development of the DISABKIDS® questionnaires actually strengthened their validity and reliability [7, 8], although some of this strength might be lost in later translations, as illustrated by the Norwegian translation and the results presented in this study. Comparing Swedish, Danish, and Norwegian versions using the same statistical methods could reveal any differences produced by the translation procedures.

The targeting, or extent to which items match the study population, in this study is imperfect. Item maps are provided in the Additional file 1 and show the distribution of participants with participants at the lower end of the latent variable scale and item thresholds at the high end. The consequence of this mismatch between participants and items is that estimates of person parameters are biased and standard errors of measurement are relatively large compared with those that would have been obtained if items had been better targeted. As a result, we propose to use the total score as a measure of independence because the total score, by definition, is an unbiased estimate of the true score. In clinical situations, it is important that the DISABKIDS® scores are sensitive to differences and changes in HrQoL among children with numerous problems and less important that they can distinguish between high degrees and very high degrees of HrQoL among children with few difficulties. Therefore, imprecise targeting of the DISABKIDS® scales will not impair the use of DISABKIDS® questionnaires in a clinical setting.

### Strengths and limitations

This Danish versions of the DISABKIDS® questionnaires were based on well-validated questionnaires and detailed procedures of validation, and the tests of validity and reliability were carried out according to gold-standard methods for health-related questionnaires. A random sample of patients/families were approached and some declined due to lack of time, which may have produced selection bias. Other studies have demonstrated that participants in questionnaire studies often have better metabolic control and originate from higher social classes, and our participants are all from one center [36]. However, one of the advantages of conditional inference in Rasch models is that the results concerning the analysis of validity do not depend on the distribution and sampling of participants. For this reason, it is not likely that selection bias influenced the validation process. The most important limitation is the sample size. The small sample size is in line with other similar studies testing the reliability and validity of DISABKIDS® (i.e., the Norwegian and Swedish studies) but does increase the risk of not detecting DIF and local dependency (type 2 errors). However, the sample size was sufficient to detect a lack of fit to the pure Rasch model and a lack of unidimensionality. Other types of DIF and local dependency may have gone undetected and might have been found if a larger study population had been recruited. Future use of the Danish versions should take this into account and if DISABKIDS® is implemented nationally a new validity test could be applied.

## Conclusion

The Danish version of the DISABKIDS® chronic-generic and diabetes-specific modules provides essentially valid and objective measurements. The reliability of the questionnaires was adequate, but the precision of measurement was less than satisfactory at the subscale level and could be enhanced by a few adjustments. Because DISABKIDS® results are summarized in a profile consisting of measurements from eight subscales we consider the lack of precision at the subscale level to be a minor problem. Therefore, this is a useful tool in the evaluation of HrQoL in Danish patients with T1D, and we recommend its implementation in the Danish Register for Children and Adolescent Diabetes. However, before implementing annual screening with DISABKIDS® in clinics, guidelines will be required on how to handle DIF and local dependency. In addition, item six from the Independence scale should be rephrased.

## Additional file

Additional file 1: GLLRM's of the five subscales of DCGM Emotion, Social inclusion and exclusion, Physical limitation and treatment and the two subscales of DSM Impact and diabetes treatment. (DOCX 577 kb)

## Abbreviations

CLR: Conditional likelihood ratio
DCGM-37: DISABKIDS-Chronic-Generic Module
DIF: Differential item functioning
DSM-10: Diabetes-Specific Module
GLLRM: Graphical log linear Rasch model
HrQoL: Health-related Quality of Life
IRT: Item response theory
ISPAD: International Society for Pediatric and Adolescent Diabetes
T1D: Type 1 diabetes
