# Supplementary material for: Item analysis using Rasch models confirms that the Danish versions of the DISABKIDS® chronic-generic and diabetes-specific modules are valid and reliable
Source: Health Qual Life Outcomes. 2017 Mar 1;15:44. doi: 10.1186/s12955-017-0618-8 (PMC5333394; doi:10.1186/s12955-017-0618-8)
Supplement: Additional file 1: — GLLRM's of the five subscales of DCGM Emotion, Social inclusion and exclusion, Physical limitation and treatment and the two subscales of DSM Impact and diabetes treatment. (DOCX 577 kb) [file 12955_2017_618_MOESM1_ESM.docx]

Additional file 1

regarding:

# Item analysis using Rasch models confirms that the Danish Versions of the DISABKIDS® chronic-generic and diabetes-specific modules are valid and reliable.

Authors:

Julie Bøjstrup Nielsen^1,^ Julie Kyvsgaard^1^, Stine Møller Sildorf^1^, Svend Kreiner^2^, Jannet Svensson^1^

Affiliations:

1. Copenhagen Diabetes Research Center (CPH-DIRECT), Department of Paediatrics, Herlev University Hospital, Herlev Ringvej 75, 2730 Herlev, Denmark
2. Section of Biostatistics, Department of Public Health, Faculty of Health and Medical Sciences, University of Copenhagen, Øster Farimagsgade 5, 1014 Copenhagen K, Denmark

Corresponding author:

Julie Bøjstrup Nielsen

E-mail: julie.bojstrup@gmail.com

Telephone: +45-28701214

DISABKIDS® – Supplementary Information

*1 Introduction*

This supplement describes the results of analyses of DISABKIDS® items by graphical log linear Rasch models (GLLRM) (1)

Table 1.1 provides an overview of the DISABKIDS® items and the eight different subscales measuring domains related to quality of life of diabetes patients.

Table 1.1 DISABKIDS® items

| Domain | Subscales | Questions |
| --- | --- | --- |
| Mental | Independence | 1-6 |
|  | Emotion | 13-19 |
|  |  |  |
| Social | Inclusion | 26-31 |
|  | Exclusion | 20-25 |
|  |  |  |
| Physical | Limitation | 7-12 |
|  | Treatment | 32-37 |
|  |  |  |
| Diabetes module | Impact | 1-6 |
|  | Treatment | 7-10 |
|  |  |  |

Response categories: never – rarely – often – very often – always

Item scores are coded from 0 to 4 so that a total score of zero indicate no problems whereas maximum score (24 or 28) refer to severe problems.

- 1. **Item analysis by Rasch models and graphical log linear Rasch models.**

The required properties of criterion-related construct validity are: (i) unidimensionality, (ii) monotonicity, (iii) local independence, and (iv) lack of differential item functioning (DIF).

The first property, unidimensionality, means that responses to items (questions) depend on a single underlying dimension or construct (e.g. independence). The person parameter is a quantitative expression of this construct. The statistical estimates of the person parameters serve as measurements of the construct. In most applications of Rasch models, the person parameter is regarded as the outcome on a latent unobservable variable.

The second property, monotonicity, means that the *expected* item scores are monotonically increas­ing functions of the person parameter. This property implies that the total score over all items will be positively correlated to all variables, which are known to be positively correlated to the latent variable. Criterion validity requires that the total score is correlated to variables that are known in advance to be correlated to the latent variable. For this reason, evidence against criterion validity is also evidence against internal construct validity.

The third property, local independence, means that responses to item are conditionally independent given the latent variable. In other words, that the presence of the latent variable explains why responses to items are marginally dependent.

The fourth property, no DIF, means that item parameters are the same in different groups of participants (eg patient and controls, men and women, young and old, before and after treatment). DIF in items is a serious concern in all comparative studies because DIF implies that measurement and therefore comparisons can be confounded.

Finally, statistical sufficiency means that all information on the person parameter is collected in the total score over all items in the sense that the conditional distribution of item responses given the total score does not depend on the person parameter. The statistical sufficiency is the property that sets Rasch models apart from other IRT models.

**1.2 Graphical log linear Rasch models**

In health related scales, it is rare to find items that satisfy the requirements of local independence and no DIF. In such cases, one can use graphical log linear Rasch models (1–4) that relax the requirements of local independence and no DIF. Instead, these models require that local dependence and DIF is uniform in the sense that the strength of the conditional association between items and between exogenous covariates is the same for all persons irrespectively of the value of the person parameter. Apart from the relaxation of the requirements of no local independence and no DIF, these models share all the other properties of Rasch models including a sufficient person score and the possibilities of conditional inference. The measurements by items from such items is essentially valid and objective (1).

**1.3 Item analysis by Rasch models**

The contents of a careful item analysis by Rasch models is discussed by others (5). The Rasch analysis may be illustrated with different techniques, but they address the same issues that we address. In addition to a careful appraisal of internal construct validity by an assessment of the adequacy of the Rasch model that address all the properties that items from Rasch models are supposed to exhibit, the analysis should also evaluate the degree to which the scale is appropriate for the study population by assessm­ent of reliability, targeting and measurement error.

**1.4 Assessing the adequacy of the Rasch model**

To take advantage of the possibility of avoiding assumptions on the distribution of the latent variable, the analysis in this study was based on the principles for conditional inference proposed and motivated by Rasch (6) and subsequently developed in a number of papers [7,8,9] . Our analysis assessed the overall fit of the model and the over-all assessment of no DIF conditional likelihood ratio test (8). The fit of specific items to the Rasch model was assessed by conditional infits and outfits (9) and by comparison of the observed and expected correlation between scores for separate items and the summated restscore over all other items (9,10). Finally, the assumptions of local dependence and no DIF was tested by the conditional likelihood ratio tests (2) and by the analyses of the partial association of items and exogen­ous variables given total score over other items (11).

**1.5 Technical properties: reliability and targeting**

Item analysis by Rasch models attempts to assess whether summated scales are valid and objective, but it is also necessary to assess whether the scale is useful in the populations that it is meant for. For this reason, we also assess the reliability and the degree to which items can be said to target the population of patients.

Classical test theory (CTT) defines the reliability of a summated score as the ratio between the variance of the true score and the variance of the observed score where the true score is the expected score. CTT assumes that the variance of the observed score is the sum of the variance of the true score and the square of the standard error of measurement. It is customary to assess the reliability of measurement by Cronbach’s α because α provides a lower bound to the true reliability if items are locally independent. We remind the reader that Cronbach’s α is not a general measure of reliability of measurement, but only a measure of reliability in the specific study population because the variance of the true score is the variance in the sample of persons. Since we expect some items to be locally dependent we use another method to calculate an unbiased measure of the true reliability under both Rasch models and log linear Rasch models (12).

The standard error of the estimate of the person parameter in Rasch models is often described as the standard error of measurement (SEM) because the person parameter estimate is regarded as a measure of the latent trait on which it depends. Calculation of SEMs is described in Kreiner & Christensen (13). These methods can easily be modified to calculate the SEM of any function of the person estimates inclusive the function describing how the true (expected) score depends on the person. In this paper, we use this method to calculate the SEM of the total scores regarded as estimates of the true scores.

It is a fundamental property of Rasch models that person parameters and item thresholds have values on the same parameter scale, and targeting is usually assessed in an informal way by so-called items maps where the distribution of persons is compared to the distributions of items. Good targeting does not require that the distributions are exactly the same, but the range of the majority of the persons should be included in the range of the item parameters and the distribution of the item thresholds should not be too skewed leaning towards either low or large person parameter values.

Since it is known that person parameter estimates are biased with very large standard errors of measurement we supplement the item maps with calculation of the average bias and the average standard errors of measurement in the study population (13).

**1.6 Overview of results included in the supplement**

The purpose of this supplement is to describe the GLLRMs for each of the different DISABKIDS® domains. The description includes the following information for each of the DISABKIDS® subscales:

1. A so-called Item response theory (IRT) graph showing relationships between the items, the latent variable, that items are supposed to measure (in this case HrQoL) and the background variables. The IRT graph include information on local dependence, differential item functioning (DIF) and the effect of background variables on HrQoL .
2. The global tests of homogeneity and no signs of DIF, demanded to fulfil the pure Rasch. Homogeneity is tested using the conditional likelihood ratio (CLR) test proposed by Andersen (8) The hypothesis is that item parameters are the same for children with a high score and children with a low score. Likewise CLR is used to test for DIF, here taking the stand that item parameters are the same in groups of background variable such as e.g. gender and age groups.
3. The test to show if the GLLRM is supported using Kelderman’s CLR test (2). This test identifies signs of local dependency (LD) and DIF, which is accepted in the GLLRM as opposed to the pure Rasch model.
4. The test for Item fit statistics. This test supports the claims that the separate items fit the GLLRM, which is seen in the IRT graph.
5. The estimates of the item parameters of the GLLRM.
6. The standard errors of the total score; that is the estimates of the true expected score. This is relevant if the total score is the preferred measure of HrQoL. Measurement by items in a GLLRM can be given either by estimates of person parameters or by the sufficient raw score.
7. Table showing the so-called DIF equated scores that show how scores should be adjusted to make them comparable across groups with differentially functioning items(1)
8. Table showing the effect of background variables on the total scores. If there is DIF relative to background variables, the tables also include information on the effect of the background variables on the DIF equated scores.
9. Item maps comparing the distribution of person parameters and the distribution of item parameters. This is to assess the degree to which items target the study population. Targeting is good, if the distribution of item parameters cover the range of persons without being skewed towards either low or high values of the person parameters.
10. A table providing evidence on the performance of the scale in the study population including estimates of reliability and estimates of the average standard error of measurement in the population.

**1.7 Summary of results**

The analysis rejected the fit of items to Rasch models because there was local dependence (LD) and/or differential item functioning (DIF), but found no evidence against the fit to GLLRMs where it is possible to adjust measurement for LD and DIF. Despite the lack of fit to the Rasch models, the analysis therefore supports the claims that DISABKIDS® measurement of the eight DISABKIDS® domains is essentially valid and objective.

Reliability of measurements by DISABKIDS® subscales are generally adequate. The standard errors of measurement though are high meaning that the DISABKIDS® score is not very precise. One of the reasons for this imprecision is that the study population is selected with generally few problems and higher HrQoL. The total population is likely to have lower HrQoL and more problems and this would increase the precision.

In addition to these reservations, the only thing that might be of concern is that the so-called item thresholds of the Rasch model in many cases are disordered. This is regarded as a problem by many, because it indicates, that the response categories do not function as proper ordinal categories. Since the item responses in other ways fit comfortably to the measurement models, we have not rejected the fit because of disordered thresholds, but we do point out that it will be worth looking into, if the same kind of results appear the next time data on DISABKIDS® items is analysed.

*Acronyms used throughout this supplement*

CLR Conditional likelihood ratio test

DIF Differential item functioning

GLLRM Graphical log linear Rasch model

LD Local dependence

LI Local independence

PCM Partial credit model

IRT graph Item response theory graph

*2 Independence*

Figure 2.1 shows the so-called IRT graph of the GLLRM for independence. The figure shows that there is LD between CG1 and CG3 and that there is DIF of CG5 relative to Age. The graph also shows that there is significant evidence of direct effects of age, sex and HbA1c on the independence score, but no evidence of direct effects of treatment and time against the other variables. The arrows and edges between the covariates indicate that these variables are statistically associated.


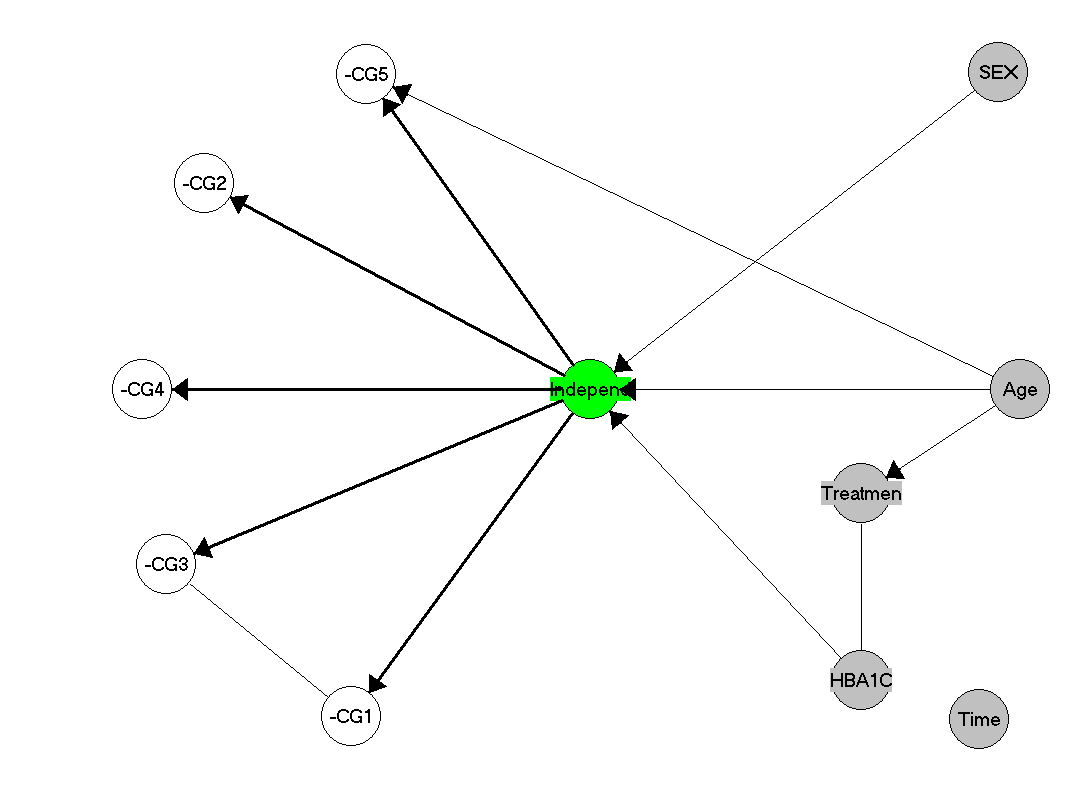


Figure 2.1. IRT graph of the GLLRM for independence

Table 2.1 shows the global CLR tests of homogeneity and no DIF. The tests are insignificant after adjustment for multiple testing by the Benjamini-Hochberg procedure (14). Table 2.2 shows the CLR tests supporting the claims of LD and DIF. Table 2.3 shows the item fit statistics. Together, the evidence in Tables 2.1-2.3 supports the fit of the items to the GLLRM. It is particular important to notice that there is no evidence of DIF relative to time (inclusion or follow-up)

Table 2.1. CLR test of homogeneity and no DIF.

CLR df p

----------------------------

Homogeneity 34.3 33 0.407

HBA1C 90.0 66 0.026

Treatment 49.8 33 0.030

Age 65.3 44 0.020

SEX 36.6 33 0.306

Time 35.5 33 0.350

Table 2.2 Evidence of LD and DIF in the independence items

| Type | Item | Item/Covariate | CLR | df | p |
| --- | --- | --- | --- | --- | --- |
| LD | CG1 | CG3 | 35,7 | 16 | 0.0032 |
| DIF | CG5 | Age | 23,4 | 8 | 0.0029 |

Table 2.3. Item fit statistics: (test for unidimensionality) Observed and expected correlations

between items and rest-scores without the items.

Item observed expected sd p

------------------------------------------------

A - CG1 0.559 0.630 0.062 0.24644

B - CG2 0.646 0.693 0.058 0.40764

C - CG3 0.744 0.619 0.064 0.05119

D - CG4 0.701 0.701 0.052 0.99789

E - CG5 0.744 0.668 0.056 0.17409

------------------------------------------------

Critical levels adjusted by the Benjamini-Hochberg procedure:

* < 5 % FDR, ** < 1 % FDR, *** = FDR < 0.1 % FDR

Table 2.4 shows the estimates of the item parameters. Because of the local dependence between CG1 and CG3, the two items have to be collapsed, so instead of possible score is 0-4 for CG1 and 0-4 for CG3, the joined score is 0 to 8, that is 9 thresholds. The thresholds of the composite item are also included in Table 2.4. Since CG5 has DIF relative to age, this item has three sets of PCM thresholds. Finally, unobserved categories are treated as If they do not exist. The missing threshold is indicated by ‘.....’.

It is often a concern if thresholds of locally independent items are disordered because it may indicate that there are problems with the ordinal structure of response categories. This is the case for CG5 in age group 8-12, where the third threshold is much larger than the fourth.

Table 2.4. Estimates of item parameters. Unobserved categories are treated as If they do not exist, in which case the missing thresholds are indicated by ‘-----’. In formal terms, this means that thresholds are equal to minus infinity. Disordered thresholds are written with bold face letters.

Item Thresholds

------------------------------------------------------------------

CG2 ----- -4.24 0.24 2.25

CG4 -3.45 -0.27 0.93 2.30

CG5

Age = 8 – 12 **0.86 -0.91** 0.34 1.27

Age = 13 – 14 ----- ----- 0.96 1.49

Age = 15 - 17 -2.99 -1.33 0.78 3.00

A-CG1 & C-CG3

----- -4.36 -3.94 -1.00 0.32 1.49 2.30 5.21

------------------------------------------------------------------

Measurement of the independence of the children may either consist of estimates of the person parameters of the log linear Rasch model or of the observed scores regarded as estimates of the true scores where LD and DIF is taken into account.

Standard errors of measurement can be calculated for both the estimates of the person paramet­ers and for the observed scores. In daily practice the calculation of the person parameter is not practical, therefore we provide standard errors for the observed scores. The DIF among the items relative to age implies that measurement may work differently in the different age groups. For this reason, measurement errors have to be calculated separately for each age group as seen in table 2.5.

During statistical analyses comparing the general level of independence in different age groups, results will be confounded because of the DIF of CG5 relative to age. To take DIF into account, we must either base analyses on the estimates of the person parameters, or we should adjust the scores for some age groups so that they are comparable to a reference age groups. Table 2.6 shows how to adjust scores to make them comparable to scores from the youngest age group. Little adjustment is needed if the score is less than eight or larger than 17, but considerable adjustment is required for scores between 8 and 16. A total score of 10 in age group 13-14 should, for instance, be increased to 11.39 to be comparable to a total score in age group 8-12.

Table 2.5. Standard errors of the observed score regarded as estimates of the true scores.

AGE

score 8-12 13-14 15-17

----------------------------

2 0.01 0.01 0.01

3 0.91 0.95 0.93

4 1.09 1.26 1.16

5 1.05 1.43 1.21

6 1.00 1.45 1.19

7 1.20 1.31 1.19

8 1.47 1.11 1.25

9 1.69 1.14 1.33

10 1.81 1.29 1.42

11 1.84 1.43 1.49

12 1.80 1.53 1.54

13 1.73 1.59 1.56

14 1.66 1.61 1.55

15 1.57 1.57 1.51

16 1.46 1.47 1.41

17 1.29 1.31 1.27

18 1.05 1.06 1.05

19 0.75 0.75 0.78

Table 2.6. DIF equated scores.

AGE

score 8-12 13-14 15-17

----------------------------

1 1.00 1.00 1.00

2 2.00 2.18 2.18

3 3.00 2.94 2.90

4 4.00 3.74 3.77

5 5.00 4.39 4.59

6 6.00 4.94 5.35

7 7.00 5.48 6.06

8 8.00 6.18 6.83

9 9.00 7.21 7.82

10 10.00 8.61 9.12

11 11.00 10.23 10.63

12 12.00 11.75 12.07

13 13.00 13.03 13.34

14 14.00 14.14 14.48

15 15.00 15.15 15.53

16 16.00 16.12 16.52

17 17.00 17.08 17.44

18 18.00 18.04 18.30

19 19.00 19.01 19.12

The effect of sex, age and HbA1c on the independence score is illustrated in Tables 2.7-9.

Table 2.7 shows the average independence scores among boys and girl. The difference is highly significant (p = 0.0005) with girls experiencing more problems that boys.

Table 2.7. Average independence scores among boys and girls.

Gender Mean se

-------------------------

Boy 14.99 0.44

Girl 12.75 0.46

-------------------------

Table 2.8 shows the average independence scores in different age groups among boys and girl. Because of the DIF relative to age, the table shows both the difference between the both observed and DIF equated scores. The number of problems is increasing with age. The effect is significant for both the observed and the adjusted scores (p = 0.003 and p = 0.004) but the effect is more pronounced on the adjusted scores.

Table 2.8. The average independence scores in different age groups.

Observed Adjusted

Age Mean se Mean se Bias

----------------------------------------------

8 - 12 15.23 0.50 15.23 0.50 0.00

13 - 14 13.56 0.62 13.29 0.74 0.27

15 - 17 12.77 0.55 12.65 0.65 0.12

----------------------------------------------

Finally, Table 2.9 shows the effect of HbA1c on independence. The level of independence problems is increasing with HbA1c. The effect is significant (p = 0.015).

Table 2.9. The average independence scores in groups defined by HbA1c

HbA1c Mean se

-------------------------

30 - 55 14.79 0.60

56 - 65 14.10 0.53

66 - 89 12.55 0.71

-------------------------

Since measurement in Rasch and other IRT models depends on the value of the person parameter it is interesting to investigate how close each individuals score is to the values of person parameters where measurement precision is optimal.

It is proposed that the target of the scale is where the standard error of the person parameter estimate is smallest (9). Since there is DIF in the Independence scale, the target of the scale may be different in different age groups. This turns out to be the case, because the independence target in age group 8-12 is equal to 0.17 where the standard error of the person estimate is 0.55 and where the expected (“true”) score is equal to of 10.9. In age group 13-14, the independence target is 1.14 (se = 0.62 and true score = 13.8). In age group 15-17 target is similar to the target of age group 13-14: target = 1.0, se = 0.64, and true score = 13.8.

The scale target in a study population may be assessed in several ways (13). Targeting will good if the distribution of item thresholds matches or targets the distribution of person, therefore item maps, comparing the distribution of persons and item parameters, is recommended.

Figure 2.2 illustrates such item maps. Because the independence score depends on age, gender and HbA1c it follows that item maps must be drawn for separate groups defined by either age, gender or HbA1c. Since there is DIF relative to age it also follows that the maps for gender and HbA1c has to be drawn for different values of age, because the distribution of the item parameters depend on age. This results in a total of 18 maps (three for the age groups, six for the gender-and-age groups and nine for the groups defined by HbA1c and age). Figure 2.2 shows the item maps for the three age groups.

Figure 2.2 shows that the DIABKIDS® targets children with somewhat larger degrees of independence problems than those found in the study population. Targeting is better for the older children where the average amount of test information provided by the independence items is 79 % of the maximum obtainable information and worst for the youngest children where independence items only provide 59 % of the possible information given perfect targeting (13). The study population is, however, not much out of target. For this reason, and because the primary focus of DISABKIDS® is to assess the quality of life among children with problems, we conclude that targeting by the independence score is adequate.


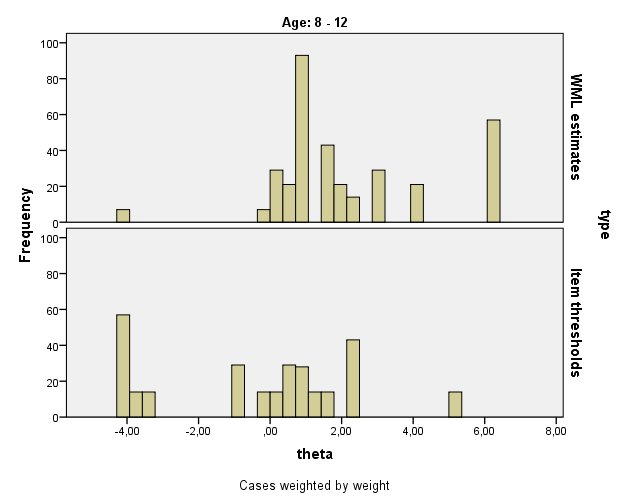

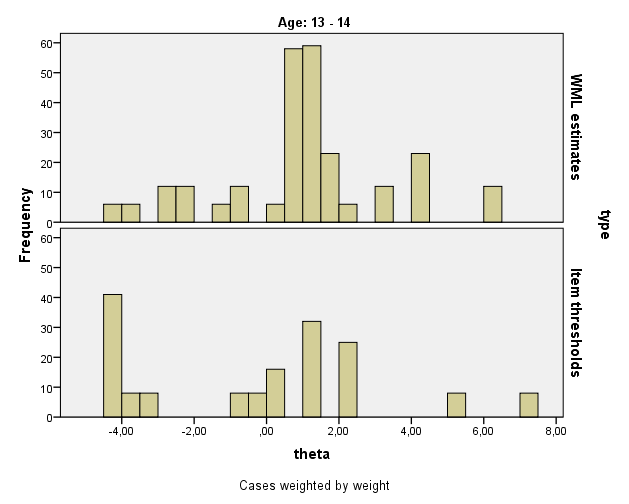


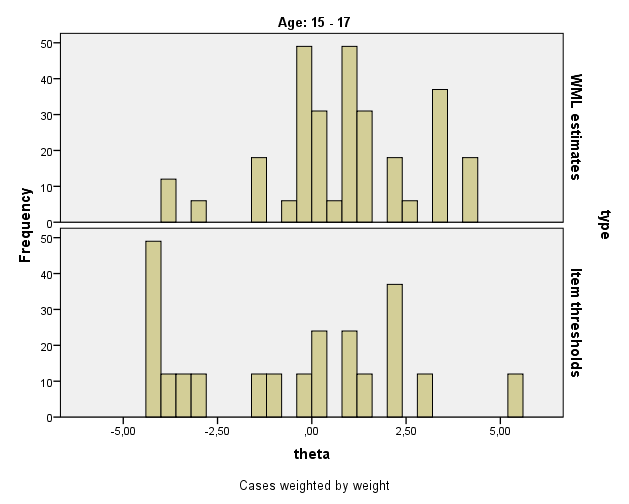


Figure 2.2. Item map showing the distribution of persons and item thresholds in different age groups.

Since measurement in practice has to be provided by the total score rather than estimates of person parameters, it is also important to assess the performance of the total score in the study population.

Table 2.10 provides this information. For each age groups, the table provides information on 1) the expected score at the target value of the independence score where test information is highest, 2) the mean and standard deviation of the observed score, 3) the reliability, 4) the scores of two randomly selected children identifies the child with most problems, and 5) the average standard error of measurement of the observed score. The table shows again, that the Independence items are somewhat out of target for the youngest children. Despite this, and the fact that measurement error is not impressive, reliability is high. We therefore conclude that the Independence scale is adequate for statistical applications, but that measurement error may be a problem in clinical applications.

Table 2.10. Assessment of targeting and reliability in subpopulations defined by age and sex

Target Population average

Age SEX n Theta SEM TS SEM Theta SEM TS SEM rel.

--------------------------------------------------------------------------------

8 - 12 Boy 25 0.17 0.54 10.86 1.84 2.74 0.91 16.08 1.17 0.89

13 - 14 Boy 21 1.14 0.62 13.78 1.61 1.34 0.80 14.05 1.32 0.87

15 - 17 Boy 23 1.03 0.64 13.20 1.56 1.81 0.77 14.65 1.34 0.83

8 - 12 Girl 23 0.17 0.54 10.86 1.84 1.42 0.68 14.30 1.55 0.71

13 - 14 Girl 22 1.14 0.62 13.78 1.61 0.86 0.82 13.09 1.28 0.91

15 - 17 Girl 24 1.03 0.64 13.20 1.56 -0.08 0.73 10.96 1.40 0.82

Table 2.11. Assessment of targeting and reliability in subpopulations defined by HbA1c and age

Target Population average

HBA1C Age n Theta SEM TS SEM Theta SEM TS SEM rel.

--------------------------------------------------------------------------------

30 – 55 8 - 12 15 0.17 0.54 10.86 1.84 2.57 0.93 15.53 1.16 0.92

56 – 65 8 - 12 21 0.17 0.54 10.86 1.84 1.70 0.74 14.76 1.45 0.79

30 – 55 13 - 14 12 1.14 0.62 13.78 1.61 2.25 0.81 15.92 1.31 0.75

56 – 65 13 - 14 14 1.14 0.62 13.78 1.61 0.66 0.80 12.79 1.31 0.90

66 – 89 13 - 14 12 1.14 0.62 13.78 1.61 0.16 0.83 11.83 1.25 0.93

30 – 55 15 - 17 15 1.03 0.64 13.20 1.56 1.07 0.75 13.13 1.37 0.87

56 – 65 15 - 17 13 1.03 0.64 13.20 1.56 1.74 0.77 14.46 1.33 0.85

66 – 89 15 - 17 17 1.03 0.64 13.20 1.56 0.11 0.74 11.29 1.37 0.87

*3 Emotion*

We refer to the section on independence for the general comments relating to the methods. This section therefore only include comments specific to the measure of emotion.

Figure 3.1 shows the GLLRM. There is DIF relative to sex and age, but no local dependence. The total score over emotion items depends on Age and Sex. Tables 3.1-3.3 provide the evidence supporting the GLLRM defined by Figure 3.1.


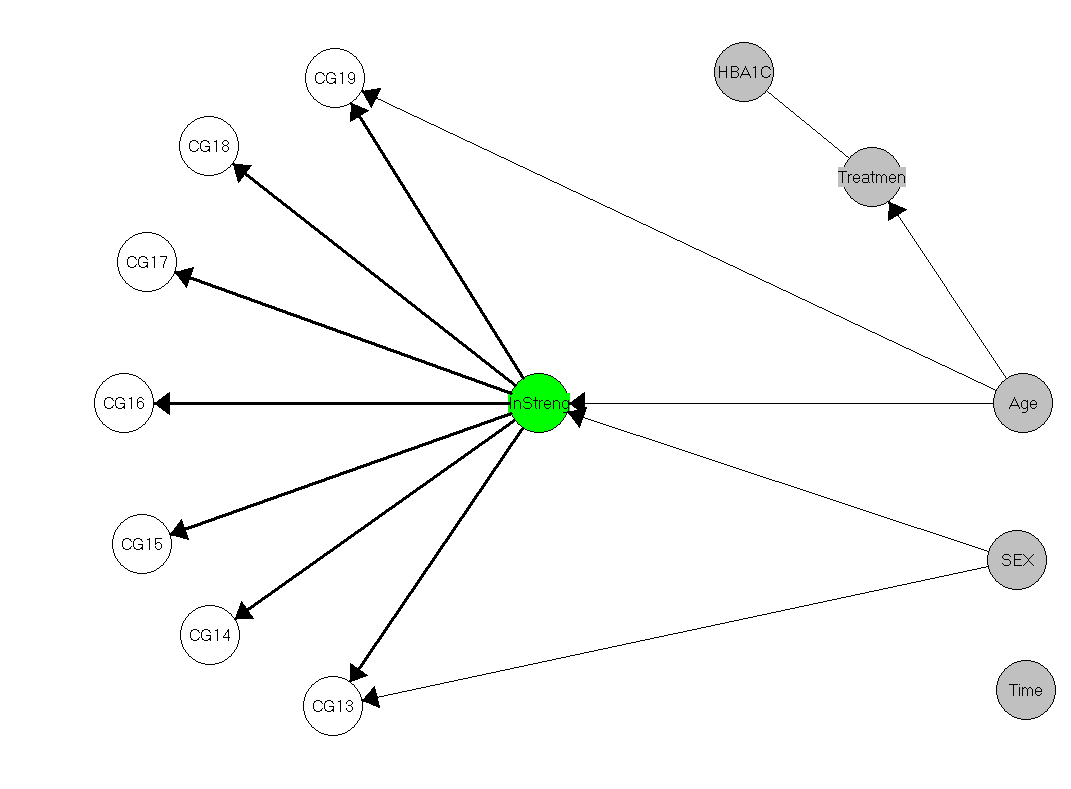


Figure 3.1. IRT graph of the GLLRM for emotion

Table 3.1. CLR test of homogeneity and no DIF.

CLR df p

----------------------------

Homogeneity 35.3 33 0.360

HBA1C 72.3 66 0.277

Treatment 34.4 33 0.399

Age 66.4 46 0.026

SEX 39.1 27 0.062

Time 26.9 33 0.765

Table 3.2 Evidence of LD and DIF in the emotion items

| Type | Item | Item/Covariate | CLR | Df | p |
| --- | --- | --- | --- | --- | --- |
| DIF | CG14 | Sex | 14.4 | 4 | 0.0062 |
| DIF | CG19 | Age | 26.1 | 8 | 0.0010 |

Table 3.3. Item fit statistics: Observed and expected correlations

between items and rest-scores without the items.

Item observed expected sd p

------------------------------------------------

CG13 0.657 0.545 0.078 0.14633

CG14 0.602 0.588 0.065 0.83090

CG15 0.501 0.587 0.065 0.18575

CG16 0.623 0.580 0.064 0.50293

CG17 0.628 0.580 0.066 0.46789

CG18 0.559 0.526 0.081 0.68415

CG19 0.551 0.573 0.062 0.72024

------------------------------------------------

Critical levels adjusted by the Benjamini-Hochberg procedure:

* < 5 % FDR, ** < 1 % FDR, *** = FDR < 0.1 % FDR

Table 3.4. Estimates of item parameters. Unobserved categories are treated as If they do not exist, because. Thresholds indicated by ‘-----’ are equal to minus infinity. Disordered thresholds are written with bold face letters.

Item Thresholds

-----------------------------------------

CG13

Sex = Boy ----- ----- ----- 0.94

Sex = Girl ----- -1.05 -0.86 0.69

CG14 -0.83 -0.79 -0.32 2.36

CG15 **-0.30 -1.29** -0.03 2.76

CG16 -1.40 -0.73 0.11 3.15

CG17 **0.05 -1.44** -0.36 1.57

CG18 ----- ----- -1.48 0.62

CG19

Age = 8-12 -0.96 **0.12 -0.46** 1.01

Age = 13-14 ----- ----- 0.54 2.71

Age = 15-17 ----- -0.52 -0.28 3.17

------------------------------------------

There are three examples of disordered thresholds in Table 3.4, indicating that the ordinal structure of response categories is less than perfectly defined. Some of these may be random error because the extreme response categories are rarely used. Since the model is accepted, we proceed as if it is not a problem for measurement, but it will be important to see whether the problem reappears, the next time data on these items are collected.

Table 3.5. Standard errors of the observed score regarded as estimates of the true scores.

DIF sources:

y = Age: 1 = 8-12 2 = 13-14 3 = 15-17

z = SEX: 1 = Boy 2 = Girl

yz yz yz yz yz yz

score 11 21 31 12 22 32

-------------------------------------------------

3 1.02 1.07 0.00 0.00 0.00 0.00

4 1.49 1.56 1.03 1.01 1.05 0.00

5 1.86 1.94 1.50 1.45 1.51 1.02

6 2.17 2.24 1.87 1.77 1.85 1.45

7 2.42 2.48 2.17 2.03 2.11 1.78

8 2.63 2.67 2.42 2.24 2.31 2.03

9 2.78 2.81 2.62 2.40 2.46 2.22

10 2.89 2.90 2.75 2.52 2.57 2.37

11 2.95 2.95 2.84 2.61 2.63 2.48

12 2.96 2.96 2.87 2.66 2.66 2.54

13 2.94 2.92 2.86 2.68 2.66 2.57

14 2.88 2.84 2.80 2.68 2.62 2.56

15 2.79 2.73 2.70 2.64 2.56 2.52

16 2.68 2.59 2.58 2.58 2.47 2.45

17 2.56 2.42 2.43 2.50 2.35 2.36

18 2.41 2.25 2.26 2.39 2.22 2.24

19 2.26 2.07 2.09 2.26 2.08 2.10

20 2.10 1.90 1.92 2.12 1.93 1.95

21 1.93 1.76 1.77 1.96 1.79 1.80

22 1.77 1.64 1.63 1.80 1.67 1.66

23 1.61 1.54 1.51 1.64 1.56 1.53

24 1.47 1.45 1.41 1.49 1.45 1.41

25 1.33 1.34 1.30 1.34 1.34 1.30

26 0.17 1.18 1.16 1.16 1.18 1.16

27 0.90 0.91 0.90 0.90 0.91 0.90

Table 3.6 – 3.8 shows the effect of DIF on the total score over all emotion items. The effect due to DIF relative to sex and age appears to be ignorable except for very high scores. Since very high scores are rare, we conclude that the effect of DIF on the emotion score is close to ignorable.

Tables 3.7 and 3.8 illustrate the average effects of age and sex on the level of problems related to emotion. Because of the DIF relative to both sex and age, the tables show results for both observed and DIF equated scores. In this case, there is little effect between the results of the observed scores and the results of the adjusted scores. For this reason, we recommend that this scale is used without adjustment for DIF.

Table 3.6. DIF equated scores.

DIF sources:

Age: 1 = 8-12 2 = 13-14 3 = 15-17

SEX: 1 = Boy 2 = Girl

yz yz yz yz yz yz

score 11 21 31 12 22 32

-------------------------------------------------

1 1.00 0.00 0.00 0.00 0.00 0.00

2 2.00 2.00 2.00 2.00 2.00 2.00

3 3.00 3.08 2.00 2.00 2.00 2.02

4 4.00 4.04 3.05 2.84 2.91 2.00

5 5.00 4.95 4.08 3.71 3.76 2.88

6 6.00 5.85 5.11 4.62 4.61 3.77

7 7.00 6.76 6.14 5.61 5.50 4.72

8 8.00 7.68 7.18 6.67 6.44 5.74

9 9.00 8.62 8.21 7.80 7.46 6.85

10 10.00 9.58 9.26 9.00 8.55 8.05

11 11.00 10.55 10.30 10.23 9.70 9.32

12 12.00 11.54 11.36 11.48 10.91 10.63

13 13.00 12.55 12.42 12.70 12.15 11.96

14 14.00 13.58 13.50 13.89 13.39 13.28

15 15.00 14.64 14.59 15.03 14.62 14.56

16 16.00 15.73 15.69 16.12 15.84 15.80

17 17.00 16.84 16.82 17.16 17.03 17.00

18 18.00 17.99 17.96 18.17 18.19 18.16

19 19.00 19.15 19.10 19.16 19.33 19.28

20 20.00 20.32 20.25 20.12 20.44 20.38

21 21.00 21.45 21.38 21.08 21.52 21.45

22 22.00 22.53 22.48 22.04 22.55 22.50

23 23.00 23.56 23.53 23.00 23.55 23.52

24 24.00 24.52 24.55 23.98 24.50 24.52

25 25.00 25.44 25.50 24.98 25.42 25.48

26 26.00 26.31 26.40 25.98 26.30 26.38

27 27.00 27.16 27.23 26.99 27.16 27.22

Table 3.7. Average emotion scores among boys and girls. Observed and IF equated scores. The differences are significant (p = 0.001)

Observed Adjusted

Sex Mean se Mean se Bias

----------------------------------------------

Boy 22.42 0.49 22.54 0.48 -0.11

Girl 19.97 0.58 19.98 0.63 -0.01

----------------------------------------------

Table 3.8. The average emotion scores in different age groups. The differences are significant (p = 0.016)

Observed Adjusted

Age Mean se Mean se Bias

----------------------------------------------

8 – 12 22.54 0.54 22.56 0.54 -0.02

13 - 14 21.05 0.76 21.20 0.79 -0.15

15 - 17 20.09 0.69 20.12 0.74 -0.04

----------------------------------------------

Figure 3.2 shows the item maps for groups defined by age and sex and Table 3.9 shows the result of the analysis of targeting and reliability. Targeting is poor except for girls of age 15-17, because the Emotion scale targets children with an expected score equal to 14-16 (depending on age and sex) whereas the average score in the study population is much smaller. For this reason, the average standard error of the estimate of the person parameters in the population is about twice as large as it would be for the population that the Emotion scale targets. Despite this, reliability is adequate, indicating that the Emotion scale is satisfactory for statistical applications.


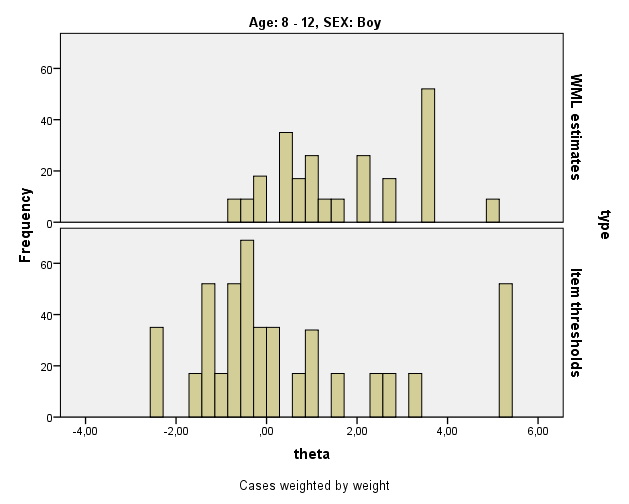

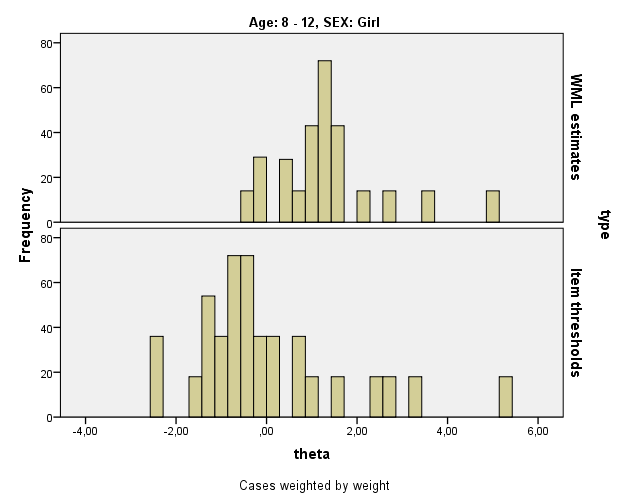


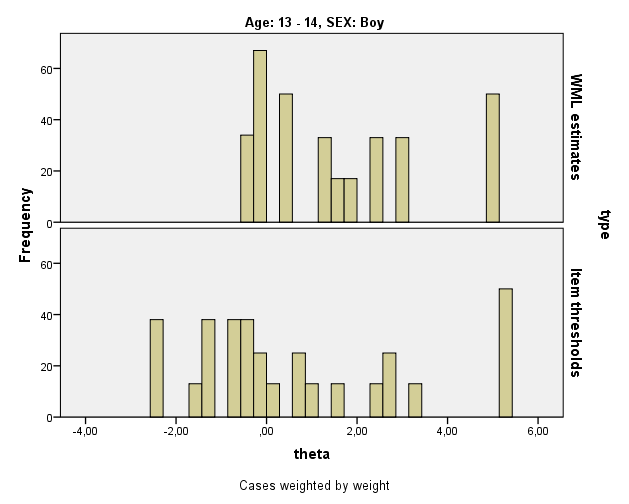

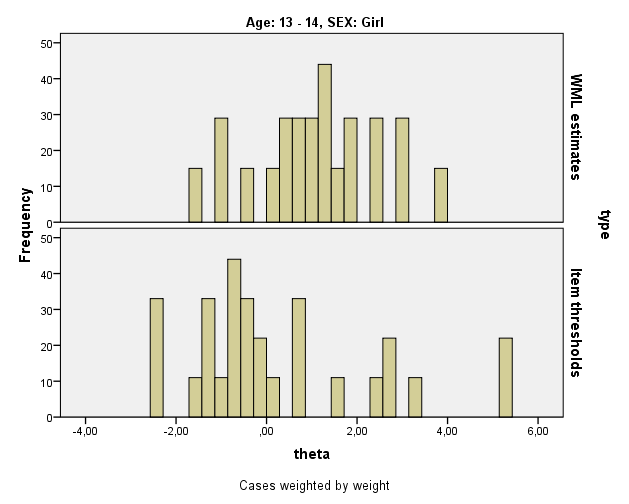


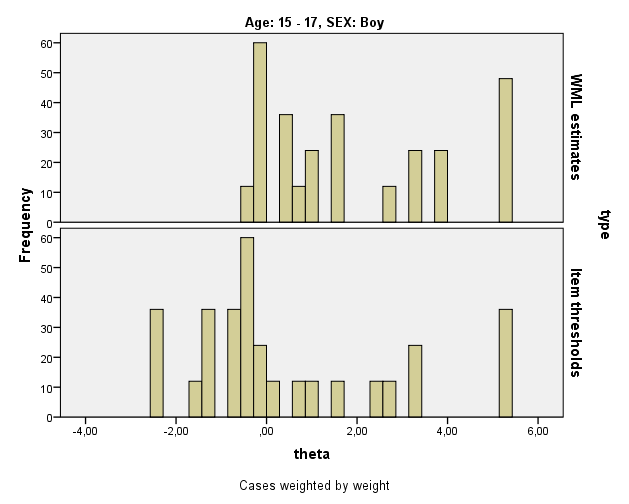

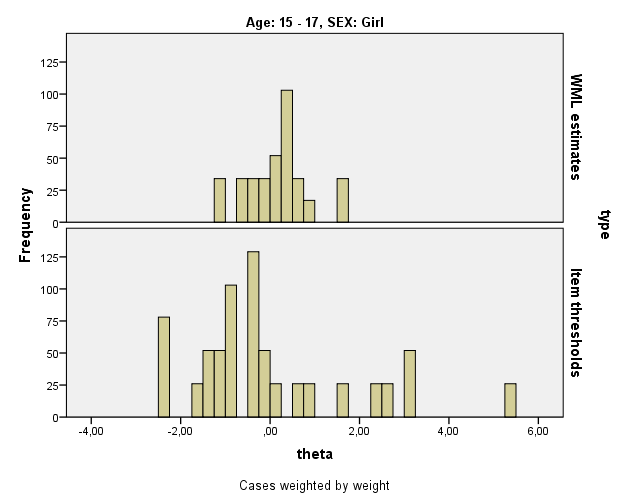


Figure 3.2 Item maps for emotion items in different groups defined by age and sex

Table 3.9. Assessment of targeting and reliability of the Emotion scale.

Target Population average

Age SEX n Theta SEM TS SEM Theta SEM TS SEM reliability

----------------------------------------------------------------------------------------

8 - 12 Boy 27 -0.69 0.34 11.86 2.96 1.67 0.66 22.74 1.63 0.83

13 - 14 Boy 20 -0.77 0.34 11.62 2.96 1.52 0.64 21.85 1.67 0.84

15 - 17 Boy 24 -0.73 0.35 12.18 2.87 1.80 0.67 22.54 1.56 0.84

8 - 12 Girl 21 -0.56 0.37 13.28 2.68 1.37 0.61 22.29 1.73 0.71

13 - 14 Girl 22 -0.75 0.38 12.38 2.66 1.07 0.59 20.32 1.80 0.88

15 - 17 Girl 22 -0.64 0.39 13.29 2.57 0.12 0.47 17.41 2.21 0.64

*4 Social inclusion*

Figure 4.1 shows the IRT graph for the GLLRM for social inclusion. Apart from the fact that two items are locally dependent, the model is a pure Rasch model. The total score over social inclusion items depends on sex, but no other variables.

The evidence supporting the model is shown in tables 4.1-4.3 and the item parameters in table 4.4. There is only one case of disordered thresholds.

*
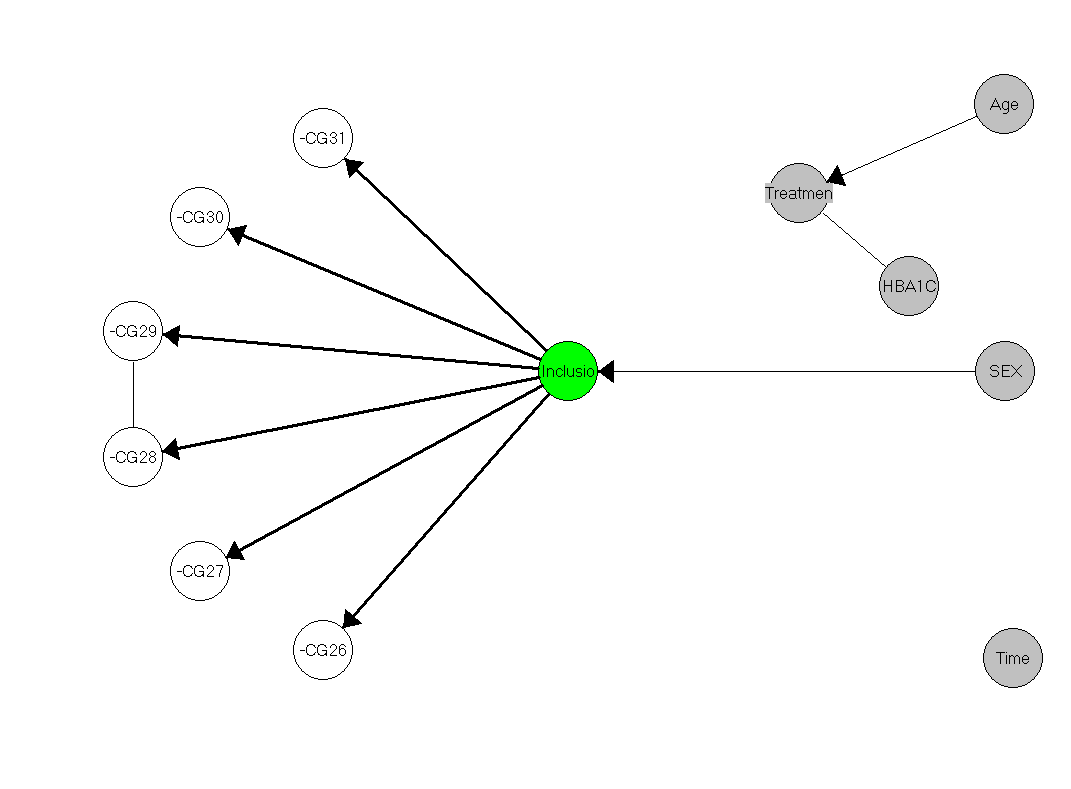
* Figure 4.1. IRT graph of the GLLRM for social inclusion

Table 4.1. CLR test of homogeneity and no DIF.

CLR df p

----------------------------

Homogeneity 25.0 28 0.627

HBA1C 75.8 56 0.040

Treatment 27.2 28 0.507

Age 70.0 56 0.099

SEX 45.2 28 0.021

Time 34.6 28 0.180

Table 4.2 Evidence of LD and DIF in the social inclusion items

| Type | Item | Item/Covariate | CLR | Df | p |
| --- | --- | --- | --- | --- | --- |
| LD | CG28 | CG29 | 53.8 | 12 | 0.0000 |

Table 4.3. Item fit statistics: Observed and expected correlations

between items and rest-scores without the items.

Item observed expected sd p

------------------------------------------------

CG26 0.425 0.349 0.081 0.35133

CG27 0.293 0.362 0.077 0.37102

CG28 0.640 0.545 0.076 0.21562

CG29 0.457 0.518 0.074 0.40817

CG30 0.533 0.324 0.094 0.02613

CG31 0.274 0.384 0.074 0.13961

------------------------------------------------

Critical levels adjusted by the Benjamini-Hochberg procedure:

* < 5 % FDR, ** < 1 % FDR, *** = FDR < 0.1 % FDR

Table 4.4. Estimates of item parameters. Unobserved categories are treated as If they do not exist, because. The missing threshold are indicated by ‘…..’.

Item Thresholds

-----------------------------------------

CG26 -1.75 -0.59 0.99 2.71

CG27 -1.73 -0.33 0.45 1.75

CG30 ----- -2.19 -0.69 0.29

CG31 **-0.12 -0.51** 1.43 1.52

CG28 & CG29

-1.19 0.40 -0.98 0.17 -0,34 0.65 0.64

------------------------------------------

Table 4.5 shows the standard errors of measurement if the score is regarded as an estimate of the true (expected) score. Measurement precision is not particular precise. Table 4.6 shows the effect of sex on social inclusion. Girls experience more problems than boys.

Table 4.5. Standard errors of the observed score regarded as estimates of the true scores.

Score SEM

-------------

3 0.95

4 1.28

5 1.53

6 1.74

7 1.95

8 2.15

9 2.32

10 2.46

11 2.54

12 2.58

13 2.56

14 2.49

15 2.39

16 2.27

17 2.14

18 2.00

19 1.86

20 1.71

21 1.52

22 1.28

23 0.94

Table 4.6 Average social inclusion scores among boys and girls.

The differences are significant (p = 0.001)

Observed

Sex Mean se

-------------------

Boy 18.27 0.39

Girl 16.38 0.43

-------------------

Figure 4.2 and table 4.7 provide information on targeting. Targeting is best for girls where the average test information is 71 % of the obtainable test information. For boys, the average test information is only 57 % of the obtainable information. The standard errors of the estimates of the person parameters are therefore larger than it would have been if the study population had been in target. The less than perfect targeting has little effect on the reliability of measurement, which is adequate in both groups.

*
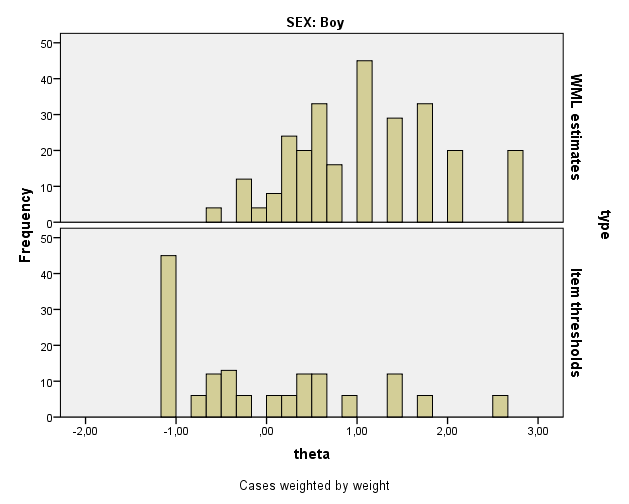

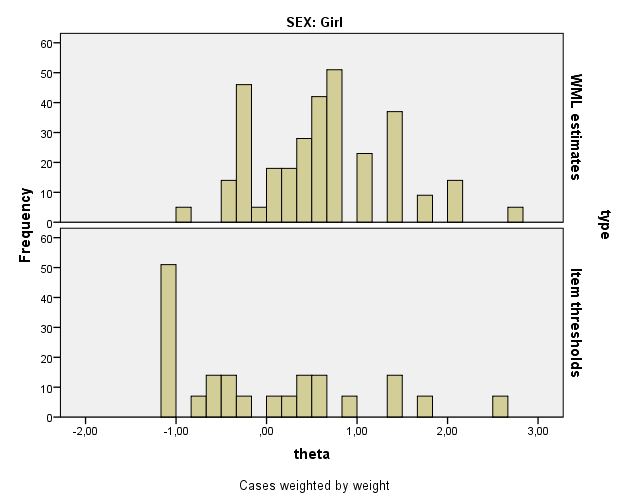
*

Figure 4.2. Item maps for social inclusion for boys and girls

Table 4.7. Assessment of targeting and reliability

Target Population average

SEX n Theta SEM TS SEM Theta SEM TS SEM reliability

-------------------------------------------------------------------------------

Boy 66 -0.21 0.39 12.12 2.58 1.07 0.55 18.27 1.91 0.63

Girl 68 -0.21 0.39 12.12 2.58 0.61 0.48 16.38 2.15 0.62

*5.* Social exclusion

Figure 5.1 shows the IRT graph of the GLLRM for social exclusion. There is no local dependence among items, but three items have DIF relative to sex, age and/or HbA1c. Sex and HbA1c are the only covariates with a direct effect on the level of problems relating to social exclusion. Tables 5.1 – 5.3 provide the evidence supporting the model. Item parameters are shown in Table 5.4. The amount of DIF and the large number of disordered thresholds is a cause for concern. Despite the fit of the social exclusion items to a GLLRM, we see the results as a challenge to the claims that these items provide valid and objective measurement.


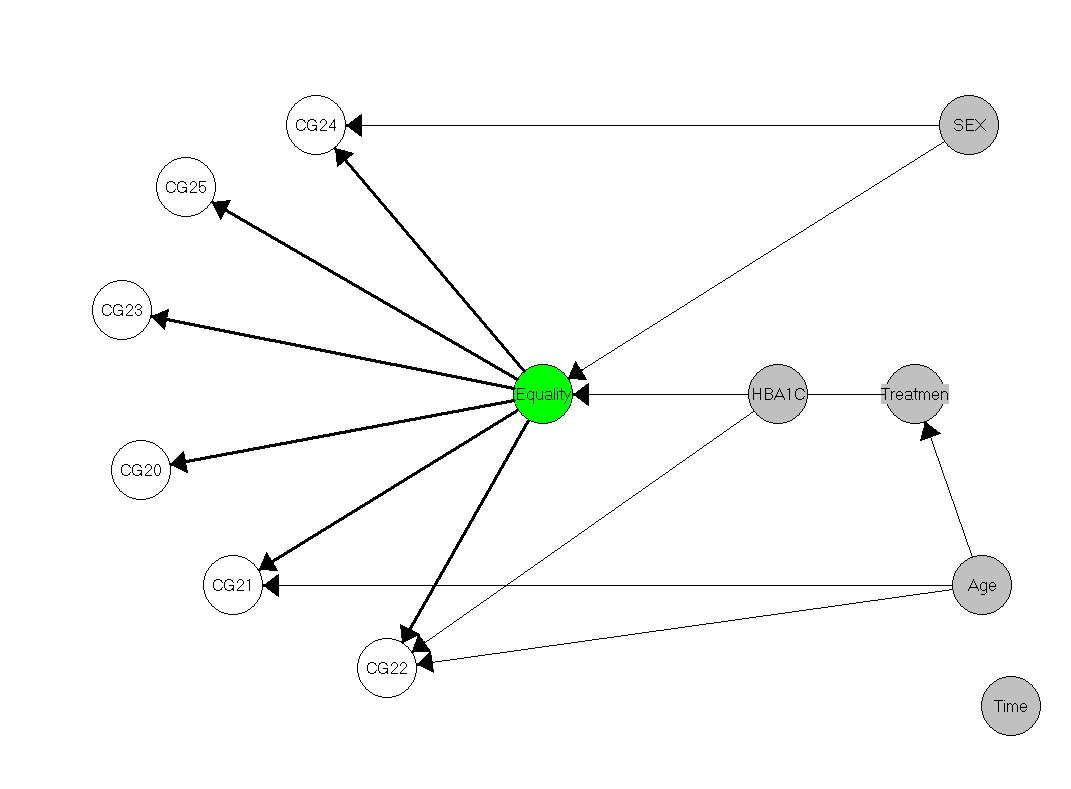


Table 5.1. CLR test of homogeneity and no DIF.

CLR df p

----------------------------

Homogeneity 44.4 42 0.370

HBA1C 96.3 68 0.014

Treatment 61.9 42 0.024

Age 58.3 46 0.105

SEX 53.2 35 0.025

Time 49.0 42 0.213

Table 5.2 Evidence of DIF in the social exclusion items

| Type | Item | Item/Covariate | CLR | df | p |
| --- | --- | --- | --- | --- | --- |
| DIF | CG21 | Age | 31.4 | 8 | 0.0001 |
| DIF | GC22 | HBA1C | 17.6 | 6 | 0.0074 |
| DIF | GC22 | Age | 22.2 | 6 | 0.0011 |
| DIF | GC24 | Sex | 12.3 | 4 | 0.0153 |

Table 5.3. Item fit statistics: Observed and expected correlations

between items and rest-scores without the items.

Item observed expected sd p

------------------------------------------------

CG20 0.647 0.506 0.094 0.13436

CG21 0.481 0.457 0.074 0.75114

CG22 0.473 0.496 0.074 0.75985

CG23 0.529 0.539 0.088 0.91428

CG24 0.428 0.527 0.076 0.19185

CG25 0.651 0.567 0.068 0.21390

------------------------------------------------

Critical levels adjusted by the Benjamini-Hochberg procedure:

* < 5 % FDR, ** < 1 % FDR, *** = FDR < 0.1 % FDR

Table 5.4. Estimates of item parameters. Unobserved categories are treated as If they do not exist, because. Thresholds indicated by ‘-----’ are equal to minus infinity.

Item Thresholds

-------------------------------------------------------------

CG20 ----- **0.34 -3.39** 0.20

CG21

Age = 8-12 -0.13  **0.81 0.36** 2.55

Age = 13-14 ----- -1.76 -0.76 1.96

Age = 15-17 -2.31 **0.45 -0.65** 0.66

CG22

Age = 8-12

HBA1C = 30 - 55 ----- **2.18 -2.37** 3.13

HBA1C = 56 - 65 ----- ----- -2.04 1.53

HBA1C = 66 - 89 ----- 3.88 -3.46 2.41

Age = 13-14

HBA1C = 30 – 55 ----- -2.71 -0.30 2.81

HBA1C = 56 - 65 ----- ----- 0.02 1.21

HBA1C = 66 - 89 ----- **-1.01 -1.41** 2.10

Age = 15-17

HBA1C = 30 - 55 ----- -1.48 1.21 2.39

HBA1C = 56 - 65 ----- ----- **1.57 0.78**

HBA1C = 66 - 89 ----- **0.22 0.14** 1.67

CG23 -2.23 **0.04 -1.55** -0.11

CG24

Sex = Boy **0.56 -0.16 -1.28** 0.11

Sex = Girl ----- -**0.52 -0.61** 1.18

CG25 **-0.34 -0.84** -0.07 2.18

-------------------------------------------------------------

The number of variables with DIF effects on social exclusion items means that tables showing standard errors of measurement and DIF equated score is too large to include in this summary and that the number of children in groups defined by these variables are too small to permit an analysis of targeting. Instead, we conclude with tables illustrating the effect of DIF equating relative to the three variables creating DIF.

Table 5.5. The average social exclusion scores in different age groups. The differences are insignificant (p = 0.12) for the observed scores, but marginally significant (p = 0.042) for the adjusted scores.

Observed Adjusted

Category Mean se Mean se Bias

----------------------------------------------

1 8 - 12 20.12 0.42 19.99 0.46 0.14

2 13 - 14 19.82 0.58 18.69 0.92 1.13

3 15 - 17 18.52 0.65 17.59 0.89 0.94

----------------------------------------------

Table 5.6. The average social exclusion scores among boys and girls. The differences are highly significant

Observed Adjusted

Sex Mean se Mean se Bias

----------------------------------------------

Boys 21.64 0.35 20.02 0.47 0.62

Girls 18.23 0.52 17.43 0.74 0.79

----------------------------------------------

The difference between boys and girls is more pronounced in the DIF adjusted scores. Note, that the fact that there is bias for both boys and girls is that sex is directly or indirectly related to other DIF sources.

Table 5.7. The average social exclusion scores in groups defined by HBA1Cs. The differences are marginally significant (p = 0.001 and 0.023 respectively)

Observed Adjusted

HBA1C Mean se Mean se Bias

----------------------------------------------

30 - 55 20.30 0.54 20.03 0.64 0.28

56 - 65 20.00 0.44 19.25 0.64 0.75

66 – 89 17.89 0.65 16.75 1.02 1.15

----------------------------------------------

Again, the effect of HbA1c is more pronounced in the DIF adjusted scores.

Finally, the relatively complicated model structure with three out of six items that function differentially relative to three different background variables means that a comprehensive analysis of targeting and reliability is impossible. Cronbach’s α is equal to 0.75 and since there is no evidence of local dependence among social exclusion items, there is little risk that this values inflates the assessment of test-retest reliability. The DIF and the fact that the social exclusion score depends on gender and HbA1c means that a proper analysis of reliability and measurement error should assess these properties in subpopulations defined by gender, age *and* HbA1c and therefore require a much larger sample than the one that has been available for this study.

*6 Physical limitation*

The model is shown in Figure 6.1. One item has DIF relative to age and the total score depends on sex and HbA1c. Tables 6.1 to 6.3 provide the evidence of the model and table 6.4 shows the estimates of the item parameters. There are several examples of disordered thresholds.
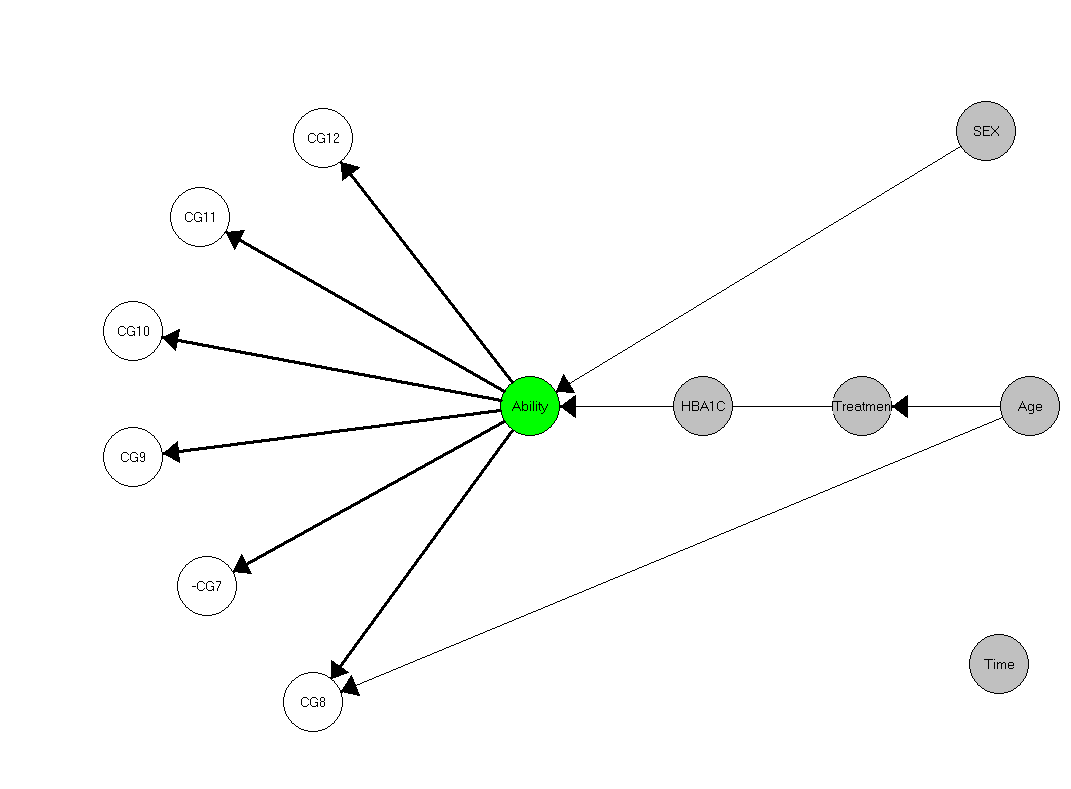


Figure 6.1. IRT graph of GLLRM for physical limitation

Table 6.1. CLR test of homogeneity and no DIF.

CLR df p

----------------------------

Homogeneity 29.2 28 0.401

HBA1C 62.5 56 0.258

Treatment 33.5 28 0.218

Age 51.8 34 0.026

SEX 40.9 28 0.055

Time 26.3 28 0.557

Table 6.2 Evidence of DIF in the physical limitation items

| Type | Item | Item/Covariate | CLR | Df | p |
| --- | --- | --- | --- | --- | --- |
| DIF | CG8 | Age | 21.8 | 8 | 0.0054 |

Table 6.3. Item fit statistics: Observed and expected correlations

between items and rest-scores without the items.

Item observed expected sd p

------------------------------------------------

CG7 0.555 0.414 0.084 0.09236

CG8 0.465 0.440 0.078 0.75049

CG9 0.491 0.457 0.078 0.66081

CG10 0.320 0.460 0.073 0.05459

CG11 0.426 0.411 0.089 0.86230

CG12 0.492 0.439 0.086 0.53427

------------------------------------------------

Critical levels adjusted by the Benjamini-Hochberg procedure:

* < 5 % FDR, ** < 1 % FDR, *** = FDR < 0.1 % FDR

Table 6.4. Estimates of item parameters. Unobserved categories are treated as If they do not exist, because. Thresholds indicated by ‘-----’ are equal to minus infinity. Disordered thresholds are written with bold face numbers.

Item Thresholds

---------------------------------------------------

CG7 ----- -0.94 -0.47 0.59

CG8

Age = 8-12 -1.35 **0.60 -1.35** 2.17

Age = 13-14 ----- -1.28 -0.42 2.72

Age = 15-17 -0.81 -0.04 0.77 3.14

CG9 -2.06 **0.40 -0.56** 1.89

CG10 -0.21 -0.10 0.37 2.19

CG11 **0.18** **-1.15**  **-1.30** 0.47

CG12 ----- -1.21 -0.51 2.36

---------------------------------------------------

Table 6.5 shows the standard errors of the score regarded as an estimate of the true expected score. Since item CG8 has DIF relative to age, it is necessary to adjust the scores, to make them compar­able across age groups. Table 6.6 shows how to do this. Below a total score of 10 there is little reason to adjust scores for children in age group 13-14. All scores in age group 15-17 has to be adjusted to make them comparable to scores in the other age groups.

Table 6.5. Standard errors of the observed score regarded as estimates of the true scores.

AGE

score 8-12 13-14 15-17

----------------------------

3 0.96 0.00 0.97

4 1.37 0.97 1.38

5 1.70 1.37 1.72

6 1.98 1.69 1.98

7 2.20 1.95 2.19

8 2.37 2.15 2.33

9 2.47 2.29 2.42

10 2.53 2.38 2.46

11 2.56 2.41 2.46

12 2.54 2.41 2.44

13 2.50 2.37 2.40

14 2.43 2.31 2.33

15 2.34 2.23 2.24

16 2.22 2.12 2.14

17 2.08 1.99 2.00

18 1.91 1.84 1.85

19 1.73 1.67 1.69

20 1.55 1.51 1.53

21 1.38 1.35 1.36

22 1.19 1.18 1.17

23 0.91 0.91 0.90

Table 6.6. DIF equated scores.

AGE

score 8-12 13-14 15-17

----------------------------

1 1.00 1.00 1.00

2 2.00 2.00 2.00

3 3.00 2.02 3.07

4 4.00 3.00 4.12

5 5.00 3.99 5.16

6 6.00 4.99 6.22

7 7.00 6.02 7.27

8 8.00 7.07 8.34

9 9.00 8.14 9.41

10 10.00 9.23 10.49

11 11.00 10.33 11.56

12 12.00 11.45 12.64

13 13.00 12.58 13.70

14 14.00 13.71 14.74

15 15.00 14.84 15.76

16 16.00 15.94 16.75

17 17.00 17.03 17.72

18 18.00 18.10 18.66

19 19.00 19.14 19.59

20 20.00 20.16 20.50

21 21.00 21.17 21.42

22 22.00 22.16 22.34

23 23.00 23.11 23.21

Table 6.7 show that there is no significant difference between ability scores in the three age groups for both observed and adjusted scores. The differences are, however, less pronounced when adjusted scores are used to compare the age groups.

Table 6.7. The average ability scores in different age groups. The differences are insignificant

Observed Adjusted

Age Mean se Mean se Bias

----------------------------------------------

8 - 12 18.60 0.51 18.60 0.51 0.00

13 - 14 17.60 0.56 17.58 0.61 0.03

15 - 17 17.53 0.49 18.13 0.24 -0.59

----------------------------------------------

Table 6.8. The average ability scores among boys and girls. The difference is significant (p = 0.002)

Sex Mean se

-----------------------

Boy 18.84 0.38

Girl 17.07 0.44

-----------------------

Table 6.9. The average ability scores in groups defined by HbA1cs. The differences are marginally significant (p = 0.011)

HBA1C Mean se

--------------------------

30 - 55 18.50 0.55

56 - 65 18.23 0.44

66 - 89 16.45 0.62

--------------------------

Tables 6.10, 6.11 and Figure 6.2 show the analysis of targeting and reliability. The analysis disregards that physical limitation depends on sex and age and therefore only consider targeting in different age groups. Again, targeting is less than perfect providing about half the average test information that could have been obtained if the study population had been in target. Measurement precision is also less than perfect, but reliability is acceptable.

Table 6.10. Assessment of targeting and reliability in groups defined by age and sex

Target Population average

Age SEX n Theta SEM TS SEM Theta SEM TS SEM reliability

----------------------------------------------------------------------------------------

8 - 12 Boy 26 -0.48 0.39 11.11 2.56 1.55 0.66 19.38 1.60 0.78

13 - 14 Boy 20 -0.54 0.41 11.36 2.41 0.96 0.58 17.90 1.80 0.65

15 - 17 Boy 22 -0.47 0.41 10.66 2.46 1.49 0.63 19.05 1.66 0.63

8 - 12 Girl 24 -0.48 0.39 11.11 2.56 0.94 0.57 17.75 1.86 0.73

13 - 14 Girl 23 -0.54 0.41 11.36 2.41 0.91 0.59 17.35 1.80 0.80

15 - 17 Girl 23 -0.47 0.41 10.66 2.46 0.59 0.50 16.09 2.07 0.53

Table 6.11. Assessment of targeting and reliability in groups defined by HbA1c and age

Target Population average

HBA1C Age n Theta SEM TS SEM Theta SEM TS SEM reliability

----------------------------------------------------------------------------------------

30 - 55 8 - 12 17 -0.48 0.39 11.11 2.56 1.13 0.61 17.88 1.76 0.84

56 - 65 8 - 12 21 -0.48 0.39 11.11 2.56 1.09 0.59 18.48 1.79 0.63

30 - 55 13 - 14 13 -0.54 0.41 11.36 2.41 1.49 0.66 19.54 1.58 0.56

56 - 65 13 - 14 14 -0.54 0.41 11.36 2.41 0.81 0.56 17.36 1.87 0.67

66 - 89 13 - 14 12 -0.54 0.41 11.36 2.41 0.35 0.53 15.33 1.98 0.81

30 - 55 15 - 17 14 -0.47 0.41 10.66 2.46 1.30 0.61 18.29 1.74 0.73

56 - 65 15 - 17 12 -0.47 0.41 10.66 2.46 1.41 0.62 18.83 1.69 0.63

66 - 89 15 - 17 17 -0.47 0.41 10.66 2.46 0.52 0.49 15.82 2.11 0.51


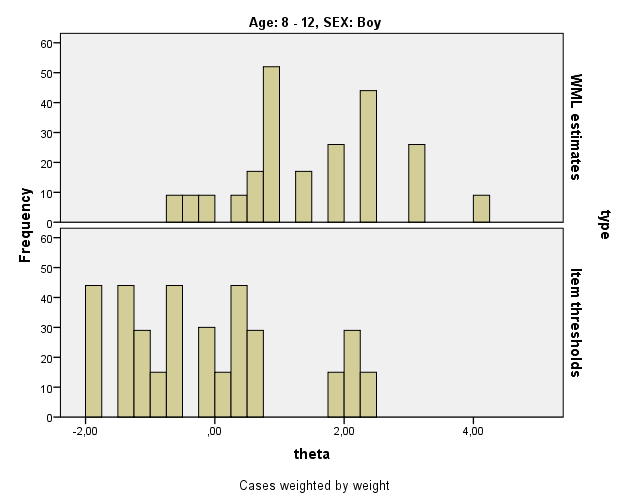

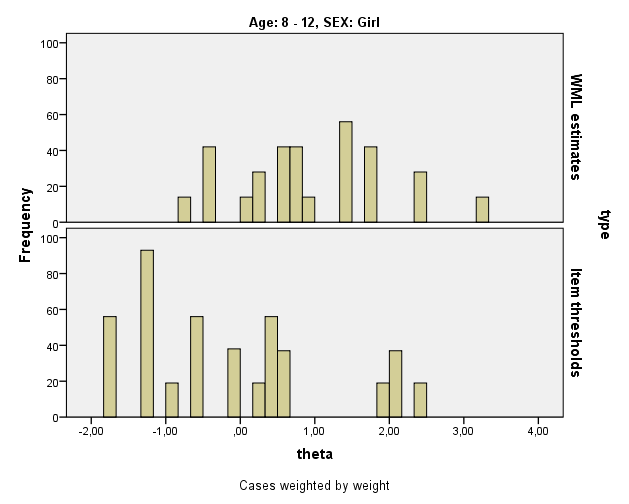


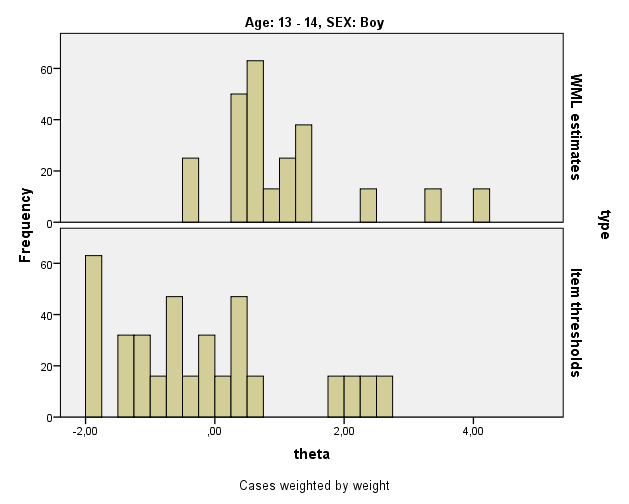

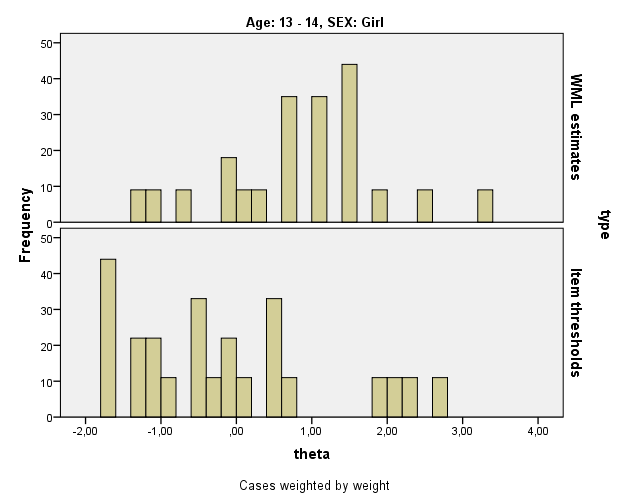


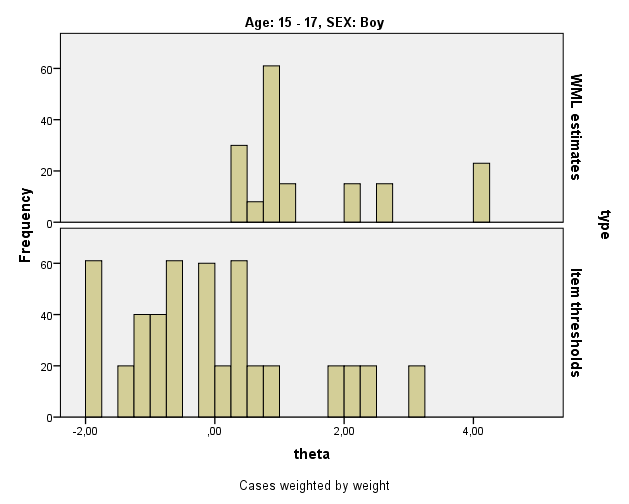

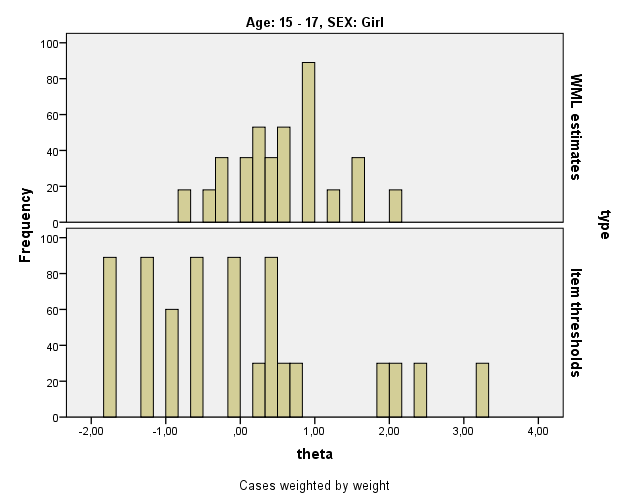


Figure 6.2. Item maps in groups defined by age and sex

*7 Treatment*

Figure 7.1 shows the model. Two items are locally dependent and treatment depends on age. Tables 7.1-7.3 provide the evidence of the model and table 7.4 shows the estimates of the item parameters. Three out of four locally independent items have disordered thresholds.

*
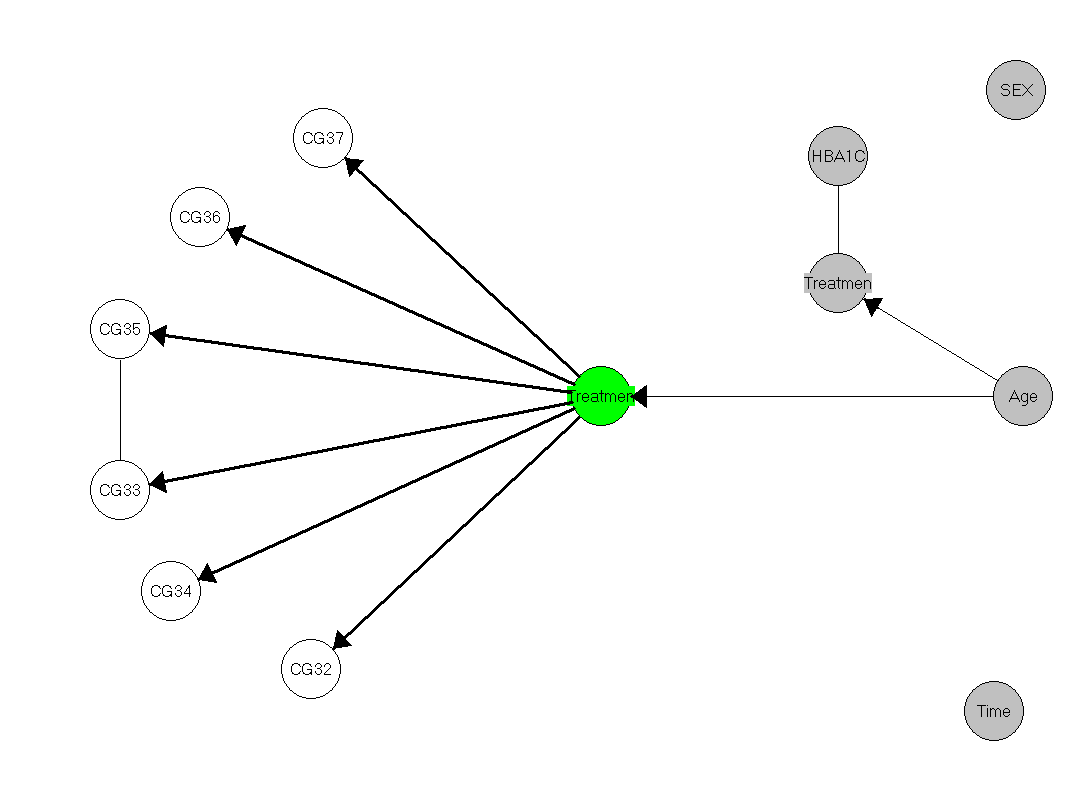
*

Figure 7.1. IRT graph of GLLRM for treatment

Table 7.1. CLR test of homogeneity and no DIF.

CLR df p

----------------------------

Homogeneity 28.8 32 0.628

HBA1C 83.0 64 0.055

Treatment 38.9 32 0.186

Age 95.4 64 0.007

SEX 34.0 32 0.372

Time 28.3 32 0.655

Table 7.2 Evidence of LD in the treatment items

| Type | Item | Item/Covariate | CLR | df | p |
| --- | --- | --- | --- | --- | --- |
| LD | CG33 | CG35 | 55.5 | 16 | 0.0000 |

Table 7.3. Item fit statistics: Observed and expected correlations

between items and rest-scores without the items.

Item observed expected sd p

----------------------------------------

CG32 0.403 0.481 0.073 0.28351

CG33 0.725 0.654 0.053 0.17948

CG34 0.392 0.468 0.083 0.36097

CG35 0.735 0.656 0.055 0.14836

CG36 0.591 0.495 0.069 0.16336

CG37 0.485 0.499 0.069 0.83841

-----------------------------------------

Critical levels adjusted by the Benjamini-Hochberg procedure:

* < 5 % FDR, ** < 1 % FDR, *** = FDR < 0.1 % FDR

Table 7.4. Estimates of item parameters. Disordered thresholds are written with bold faced numbers.

Item Thresholds

----------------------------------------------------

CG32 -0.95 -0.64 -0.57 0.48

CG34 -0.32 **0.14 -1.97** 0.08

CG36 **-0.29 -0.55** -0.55 1.21

CG37 **-0.15** **-0.57 -0.60**  1.78

CG33 & CG35

-1.31 -0.65 0.97 -1.29 0.64 0.74 1.84 2.10

-----------------------------------------------------

Table 7.5 shows the standard errors of the score regarded as an estimate of the true score.

Table 7.5. Standard errors of measurement

Score SEM

------------

1 1.05

2 1.55

3 1.93

4 2.24

5 2.48

6 2.66

7 2.80

8 2.89

9 2.94

10 2.96

11 2.95

12 2.90

13 2.83

14 2.72

15 2.59

16 2.42

17 2.23

18 2.04

19 1.86

20 1.69

21 1.53

22 1.32

23 0.98

---------------

Table 7.6. Average treatment scores in different age groups. The differences are marginally significant (p = 0.05)

Age Mean se

-------------------------

1 8 - 12 17.14 0.72

2 13 - 14 17.21 0.71

3 15 - 17 15.87 0.73

-------------------------

Table 7.7. Average treatment scores for boys and girls. The differences are insignificant (p = 0.07)

Category Mean se

-------------------------

1 Boy 17.51 0.58

2 Girl 16.02 0.60

-------------------------

Table 7.8 and Figure 7.2 show the results of the analysis of targeting and reliability. Targeting and measurement precision is poor, but reliability is adequate.

Table 7.8 Assessment of targeting and reliability in different age groups

Target Population average

Age n Theta SEM TS SEM Theta SEM TS SEM reliability

-------------------------------------------------------------------------------

8 - 12 51 -0.40 0.34 10.10 2.96 0.89 0.53 17.14 2.02 0.83

13 - 14 38 -0.40 0.34 10.10 2.96 0.82 0.52 17.21 2.08 0.77

15 - 17 40 -0.40 0.34 10.10 2.96 0.51 0.47 15.88 2.27 0.74


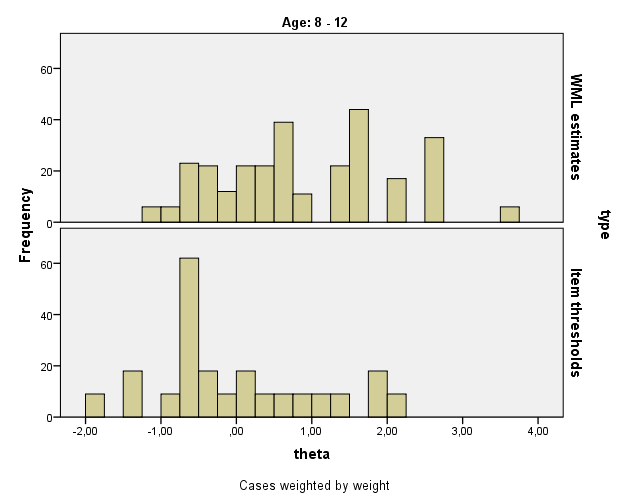

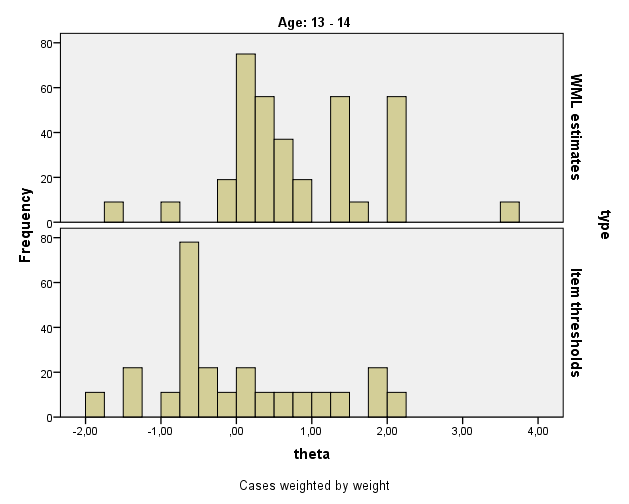


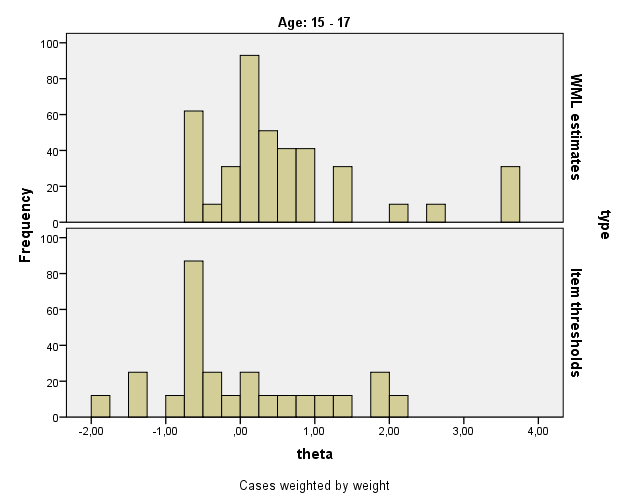


Figure 7.2 Item maps in different age groups

*8 Diabetes module: Impact*

Figure 8.1 shows the model. Two items are locally dependent and impact depends on sex and time. Tables 8.1-8.3 provide the evidence of the model and table 8.4 shows the estimates of the item parameters. Only one item with disordered thresholds.

*
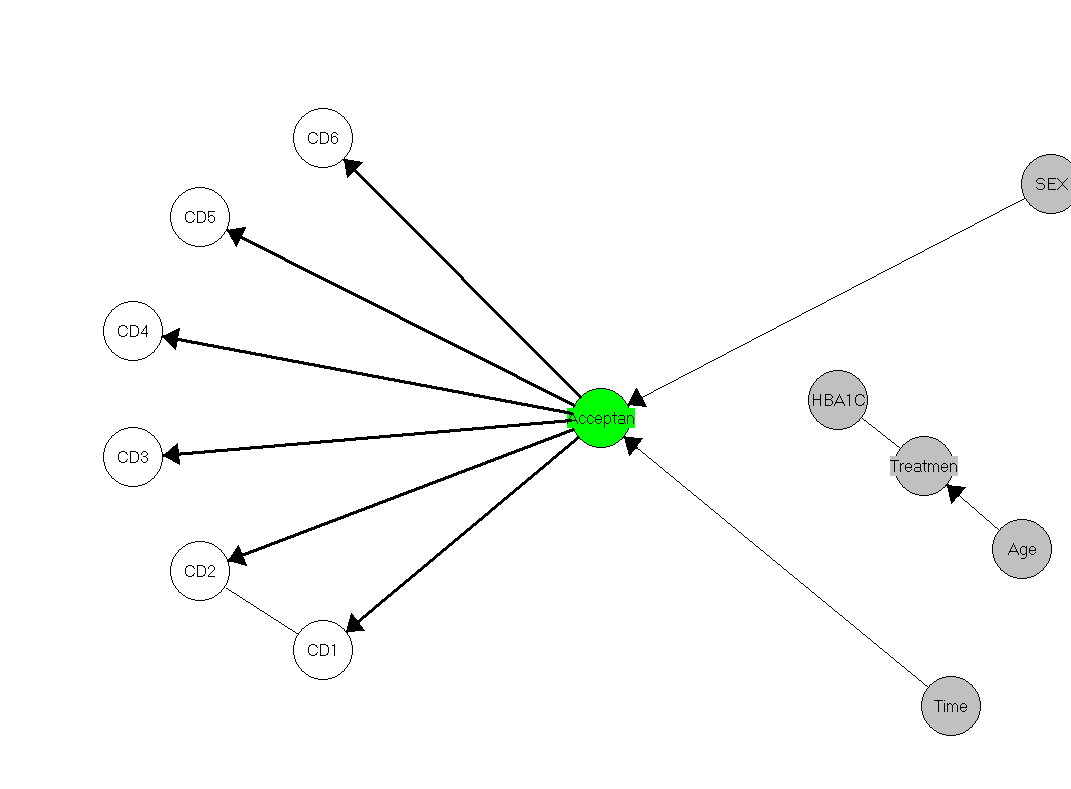
*

Figure 8.1. IRT graph of GLLRM for impact

Table 8.1. CLR test of homogeneity and no DIF.

CLR df p

----------------------------

Homogeneity 39.8 32 0.161

HBA1C 65.6 64 0.419

Treatment 42.6 32 0.099

Age 73.0 64 0.207

SEX 39.1 32 0.181

Time 36.2 32 0.280

Table 8.2 Evidence of LD in the impact items

| Type | Item | Item/Covariate | CLR | df | p |
| --- | --- | --- | --- | --- | --- |
| LD | CD1 | CD2 | 34.5 | 16 | 0.0047 |

Table 8.3. Item fit statistics: Observed and expected correlations

between items and rest-scores without the items.

Item observed expected sd p

----------------------------------------

CD1 0.578 0.557 0.076 0.78025

CD2 0.575 0.534 0.071 0.56421

CD3 0.536 0.469 0.068 0.32020

CD4 0.373 0.457 0.071 0.24027

CD5 0.500 0.468 0.070 0.64283

CD6 0.582 0.475 0.067 0.11310

-----------------------------------------

Critical levels adjusted by the Benjamini-Hochberg procedure:

* < 5 % FDR, ** < 1 % FDR, *** = FDR < 0.1 % FDR

Table 8.4. Estimates of item parameters..

Item Thresholds

----------------------------------------------------

CD3 -0.66 0.29 0.64 2.24

CD4 -2.30 0.11 0.42 2.36

CD5 **-0.16 -0.44** 1.00 2.63

CD6 -0.19 0.41 0.54 1.19

CG33 & CG35

-0.27 -1.47 0.62 -0.33 1.65 1.88

-----------------------------------------------------

Table 8.5 shows the standard errors of the score regarded as an estimate of the true score and Tables 8.6 and 8.7 shows the effect of sex and time on impact scores.

Finally, Table 8.8 shows the results of analyses of targeting and reliability.

Table 8.5. Standard errors of measurement

Score SEM

--------------

1 1.04

2 0.83

3 0.96

4 1.35

5 1.65

6 1.88

7 2.06

8 2.19

9 2.29

10 2.37

11 2.41

12 2.43

13 2.41

14 2.38

15 2.32

16 2.23

17 2.12

18 1.99

19 1.86

20 1.70

21 1.52

22 1.29

23 0.95

--------------

Table 8.6. The average impact scores in groups defined by sex. The difference is significant (p = 0.001)

Sex Mean se

--------------------------

Boy 17.28 0.48

Girl 14.96 0.54

--------------------------

Table 8.7. The average impact scores in groups defined by time. The difference is marginally significant (p = 0.046)

Time Mean se

-------------------------

Inclusion 16.69 0.46

Follow-up 15.13 0.63

-------------------------

Table 8.8. Analysis of targeting and reliability in groups defined by sex

Target Population average

SEX n Theta SEM TS SEM Theta SEM TS SEM reliability

-------------------------------------------------------------------------------

Boy 71 0.24 0.41 12.12 2.43 1.37 0.54 17.28 1.95 0.76

Girl 71 0.24 0.41 12.12 2.43 0.82 0.49 14.96 2.12 0.78


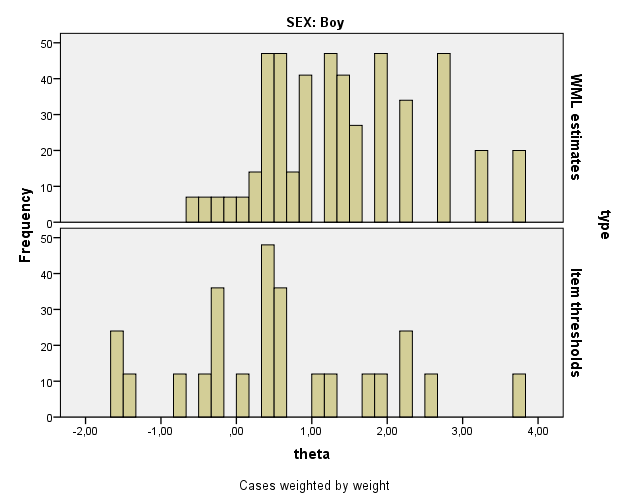

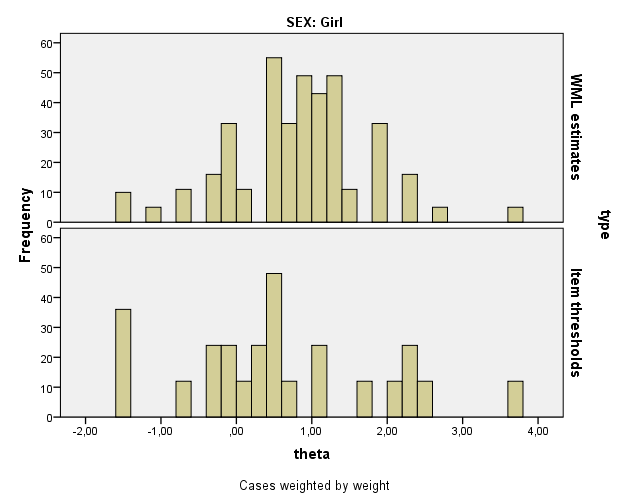


Figure 8.2 Item maps in groups defined by sex

*9 Diabetes module – treatment*

This scale contains only four items, three of which has DIF relative to age. Figure 9.1 shows the model, Tables 9.1 – 9.3 provide evidence supporting the fit to data, and Table 9.4 shows the item parameters.


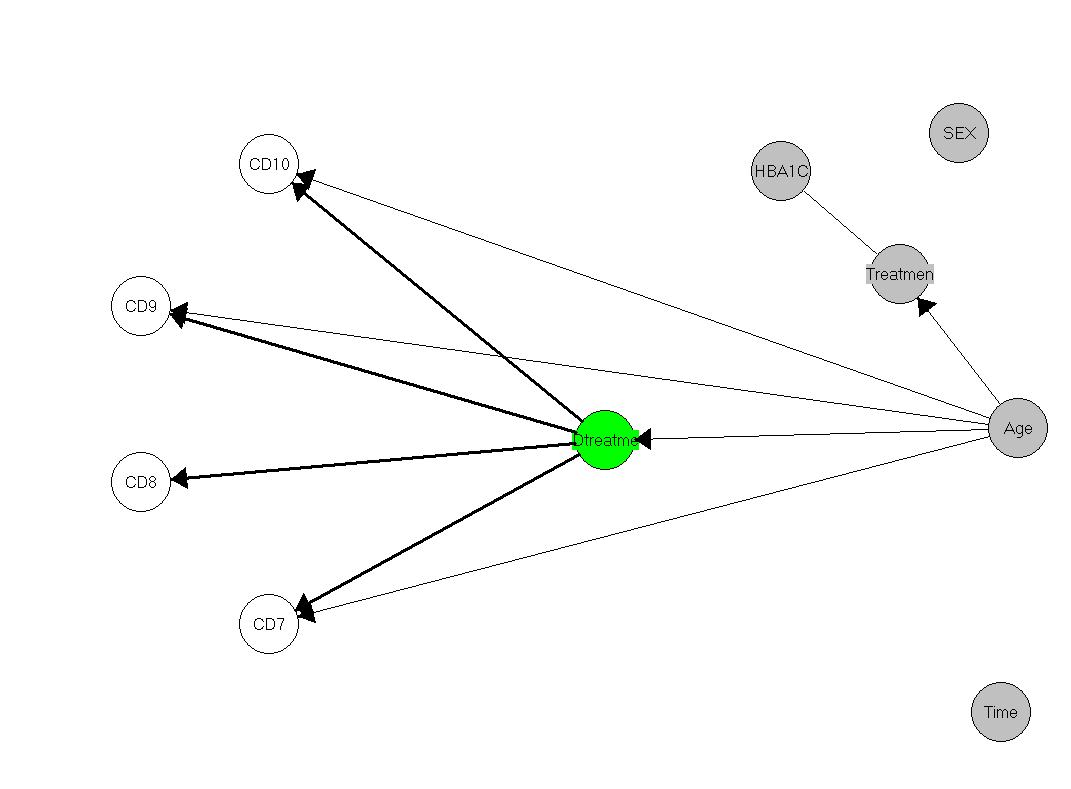


Figure 9.1. IRT graph of GLLRM for treatment

Table 9.1. CLR test of homogeneity and no DIF.

CLR df p

----------------------------

Homogeneity 26.2 38 0.923

HBA1C 107.2 76 0.011

Treatment 53.1 38 0.053

Age 7.9 6 0.249

SEX 67.7 38 0.002

Time 35.2 38 0.598

Table 9.2 Evidence of DIF in the treatment items

| Type | Item | Item/Covariate | CLR | df | P |
| --- | --- | --- | --- | --- | --- |
| DIF | CD7 | Age | 20.7 | 8 | 0.0080 |
| DIF | CD9 | Age | 26.0 | 8 | 0.0010 |
| DIF | CD10 | Age | 23.0 | 8 | 0.0034 |

Table 9.3. Item fit statistics: Observed and expected correlations

between items and rest-scores without the items.

Item observed expected sd p

----------------------------------------

CD7 0.677 0.622 0.055 0.32000

CD8 0.624 0.638 0.054 0.78449

CD9 0.638 0.655 0.050 0.72918

CD10 0.687 0.661 0.050 0.60002

-----------------------------------------

Critical levels adjusted by the Benjamini-Hochberg procedure:

* < 5 % FDR, ** < 1 % FDR, *** = FDR < 0.1 % FDR

Table 9.4. Estimates of item parameters. Threshold indicated by ‘-----’ are equal to minus infinity.

Item Thresholds

-----------------------------------------------------

CD7

age = 8 - 12 -2.06 **-0.16 -0.62** 2.15

age = 13 – 14 ----- -2.90 -0.78 4.03

age = 15 - 17 **-2.01 -2.91** -0.04 1.96

CD8 -1.22 -0.54 1.81 4.46

CD9

age = 8 - 12 **-0.90 -1.05** 0.35 0.95

age = 13 - 14 -0.89 1.32 2.17 3.22

age = 15 - 17 -0.50 -0.45 0.80 3.84

CD10

age = 8 - 12 -2.35 **-0.56 -1.20** 0.93

age = 13 - 14 **-0.69 -1.00** 0.80 2.87

age = 15 - 17 **-2.83 -2.91** 0.34 1.58

-----------------------------------------------------

Table 9.5 shows the standard errors of the observed scores. Because of the DIF relative to age, these standard errors are calculated in each age group.

Table 9.5. Standard errors of the observed score regarded as estimates of the true scores.

AGE

score 8-12 13-14 15-17

----------------------------

1 0.94 0.00 1.03

2 1.30 0.88 1.32

3 1.57 1.19 1.42

4 1.76 1.40 1.43

5 1.89 1.51 1.45

6 1.95 1.52 1.51

7 1.96 1.47 1.56

8 1.91 1.41 1.57

9 1.82 1.37 1.53

10 1.70 1.37 1.47

11 1.55 1.37 1.39

12 1.39 1.33 1.28

13 1.21 1.24 1.15

14 1.00 1.10 1.00

15 0.76 0.87 0.82

Table 9.6 shows the DIF equated scores and tables 9.7 – 9.8 shows average scores in groups defined by age and sex.

Table 9.6. DIF equated scores.

AGE

score 8-12 13-14 15-17

----------------------------

1 1.00 0.02 0.59

2 2.00 1.09 1.12

3 3.00 2.43 1.76

4 4.00 3.94 2.67

5 5.00 5.56 3.94

6 6.00 7.21 5.51

7 7.00 8.82 7.12

8 8.00 10.30 8.61

9 9.00 11.57 9.93

10 10.00 12.57 11.10

11 11.00 13.36 12.12

12 12.00 13.99 13.04

13 13.00 14.52 13.88

14 14.00 15.01 14.65

15 15.00 15.49 15.33

Table 9.7. The average treatment scores in different age groups. The differences are highly significant

Observed Adjusted

Age Mean se Mean se Bias

----------------------------------------------

8 - 12 10.55 0.48 10.55 0.48 0.00

13 - 14 9.56 0.57 11.12 0.06 -1.56

15 - 17 7.83 0.58 8.10 0.61 -0.27

----------------------------------------------

Table 9.8. The average treatment scores for boys and girls. The differences are insignificant (p = 0.79)

Sex Mean se

-------------------------

1 Boy 9.28 0.47

2 Girl 9.45 0.45

-------------------------

Finally, Table 9.9 and Figure 9.2 shows the results of the analysis of targeting and reliability

Table 9.9. Analysis of targeting and reliability in groups defined by age.

Target Population average

Age n Theta SEM TS SEM Theta SEM TS SEM reliability

-------------------------------------------------------------------------------

8 - 12 53 -0.66 0.51 6.60 1.96 0.83 0.73 10.55 1.47 0.81

13 - 14 45 -0.63 0.66 5.70 1.53 1.40 0.77 9.56 1.29 0.88

15 - 17 46 -0.24 0.64 7.68 1.57 -0.16 0.72 7.83 1.39 0.87


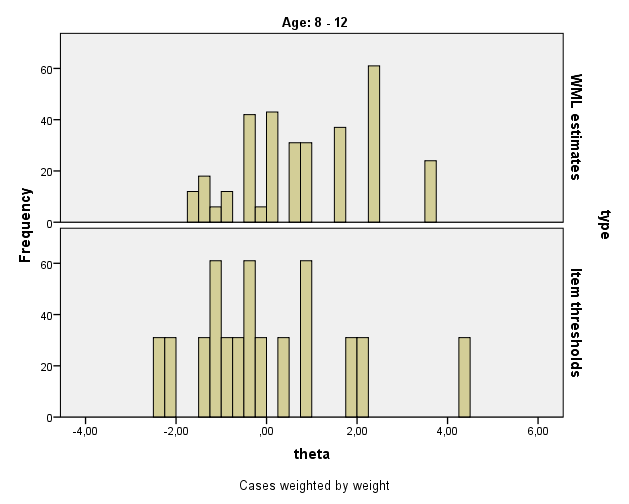

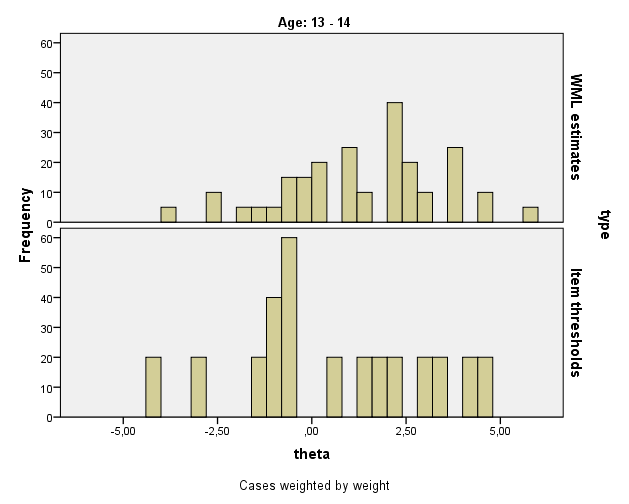


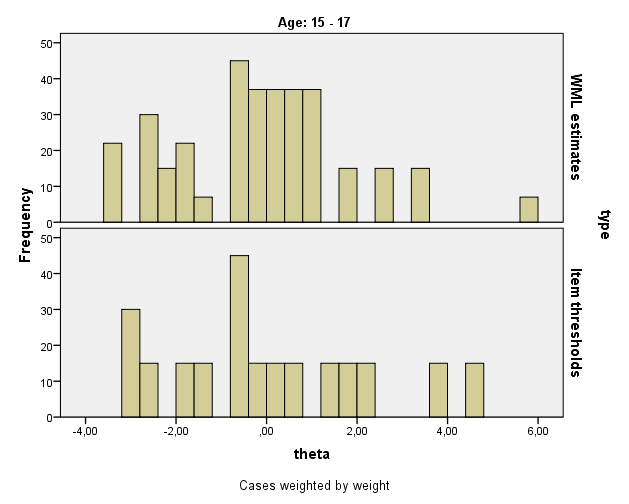


Figure 9.2 Item maps in groups defined by age.

References

1. Kreiner S, Christensen KB. Validity and Objectivity in Health-Related Scales: Analysis by Graphical Loglinear Rasch Models. In: Multivariate and Mixture Distribution Rasch Models [Internet]. Springer New York; 2007 [cited 2015 Feb 10]. p. 329–46. Available from: http://link.springer.com.ep.fjernadgang.kb.dk/chapter/10.1007/978-0-387-49839-3_21

2. Kelderman H. Loglinear Rasch model tests. Psychometrika. 1984 Jun 1;49(2):223–45.

3. Kreiner S, Christensen KB. Graphical Rasch Models. In: Mesbah M, Cole BF, Lee M-LT, editors. Statistical Methods for Quality of Life Studies [Internet]. Springer US; 2002 [cited 2015 Feb 10]. p. 187–203. Available from: http://link.springer.com/chapter/10.1007/978-1-4757-3625-0_15

4. Kreiner S, Christensen KB. Analysis of Local Dependence and Multidimensionality in Graphical Loglinear Rasch Models. Commun Stat Theory Methods. 2004 Jun;33(6):1239–76.

5. Tennant A, Conaghan PG. The Rasch measurement model in rheumatology: What is it and why use it? When should it be applied, and what should one look for in a Rasch paper? Arthritis Care Res. 2007;57(8):1358–62.

6. Rasch G. On General Laws and the Meaning of Measurement in Psychology. In The Regents of the University of California; 1961 [cited 2015 Feb 10]. Available from: http://projecteuclid.org/euclid.bsmsp/1200512895

7. Andersen EB. Asymptotic Properties of Conditional Maximum-Likelihood Estimators. J R Stat Soc Ser B Methodol. 1970 Jan 1;32(2):283–301.

8. Andersen EB. A goodness of fit test for the rasch model. Psychometrika. 1973 Mar 1;38(1):123–40.

9. Christensen KB, Kreiner S. Item Fit Statistics. In: Christensen KB, Kreiner S, Mesbah M, editors. Rasch Models in Health [Internet]. John Wiley & Sons, Inc.; 2012 [cited 2015 Feb 10]. p. 83–104. Available from: http://onlinelibrary.wiley.com.ep.fjernadgang.kb.dk/doi/10.1002/9781118574454.ch5/summary

10. Kreiner S. A Note on Item–Restscore Association in Rasch Models. Appl Psychol Meas. 2011 Oct 1;35(7):557–61.

11. Kreiner S, Christensen KB. Item Screening in Graphical Loglinear Rasch Models. Psychometrika. 2011 Mar 9;76(2):228–56.

12. Hamon A, Mesbah M. Questionnaire Reliability Under the Rasch Model. In: Mesbah M, Cole BF, Lee M-LT, editors. Statistical Methods for Quality of Life Studies [Internet]. Springer US; 2002 [cited 2015 Feb 10]. p. 155–68. Available from: http://link.springer.com/chapter/10.1007/978-1-4757-3625-0_13

13. Kreiner S, Christensen KB. Person Parameter Estimation and Measurement in Rasch Models. In: Christensen KB, Kreiner S, Mesbah M, editors. Rasch Models in Health [Internet]. John Wiley & Sons, Inc.; 2012 [cited 2015 Feb 10]. p. 63–78. Available from: http://onlinelibrary.wiley.com.ep.fjernadgang.kb.dk/doi/10.1002/9781118574454.ch4/summary

14. Benjamini, Hochberg. Controlling the False Discovery Rate: A Practical and Powerful Approach to Multiple Testing. J R Stat Soc Ser B Methodol. Vol 57:289–300.
